# Adsorption of bacteriophages on polypropylene labware affects the reproducibility of phage research

**DOI:** 10.1038/s41598-021-86571-x

**Published:** 2021-04-01

**Authors:** Łukasz Richter, Karolina Księżarczyk, Karolina Paszkowska, Marta Janczuk-Richter, Joanna Niedziółka-Jönsson, Jacek Gapiński, Marcin Łoś, Robert Hołyst, Jan Paczesny

**Affiliations:** 1 Institute of Physical Chemistry, Polish Academy of Sciences, Kasprzaka 44/52, 01-224 Warsaw, Poland; 2 Department of Molecular Biophysics, Adam Mickiewicz University in Poznań, Uniwersytetu Poznańskiego 2, 61-614 Poznań, Poland; 3 Department of Molecular Biology, University of Gdańsk, Wita Stwosza 59, 80-308 Gdansk, Poland; 4 Phage Consultants, Partyzantów 10/18, 80-254 Gdańsk, Poland

**Keywords:** Bacteriophages, Biomedical engineering, Design, synthesis and processing, Polymers, Surface chemistry, Nanobiotechnology

## Abstract

Hydrophobicity is one of the most critical factors governing the adsorption of molecules and objects, such as virions, on surfaces. Even moderate change of wetting angle of plastic surfaces causes a drastic decrease ranging from 2 to 5 logs of the viruses (e.g., T4 phage) in the suspension due to adsorption on polymer vials' walls. The effect varies immensely in seemingly identical containers but purchased from different vendors. Comparison of glass, polyethylene, polypropylene, and polystyrene containers revealed a threshold in the wetting angle of around 95°: virions adsorb on the surface of more hydrophobic containers, while in more hydrophilic vials, phage suspensions are stable. The polypropylene surface of the Eppendorf-type and Falcon-type can accommodate from around 10^8^ PFU/ml to around 10^10^ PFU/ml from the suspension. The adsorption onto the container’s wall might result in complete scavenging of virions from the bulk. We developed two methods to overcome this issue. The addition of surfactant Tween20 and/or plasma treatment provides a remedy by modulating surface wettability and inhibiting virions' adsorption. Plastic containers are essential consumables in the daily use of many bio-laboratories. Thus, this is important not only for phage-related research (e.g., the use of phage therapies as an alternative for antibiotics) but also for data comparison and reproducibility in the field of biochemistry and virology.

## Introduction

Bacteriophages are viruses that infect bacteria and are used for medical purposes since the early 1900s^1^. Despite early successes, phage therapies, i.e., curing bacterial infections with phages, were largely abandoned when antibiotics came along^2^. However, the spread of drug-resistant superbugs and lack of new antibiotics^3,4^ cause the renaissance of phage therapies^5^. Phage-based products recently reached clinical trials, e.g., curing inner ear infections^6^, urinary tract infections^7^, typhoid^8^, systemic multi-drug resistant infections^9^, or infected burn wounds (*Phagoburn* project)^10^. Among numerous trials, the level of active phages applied to patients is in the range of around 10^7^ phages/ml or lower^6,10–12^. Here, we show that in such a concentration regime, the number of active phages might decrease by many orders of magnitude depending on the properties of the container the phage suspension is stored in. Uncontrolled disappearance of phages from the suspension can cause the failure of not only phage therapies but also all other phage-based technologies, such as biocontrol applications^13^, delivery systems^14^, sensors^15^, material science^16^, or even studies on eukaryotic viruses using phage models (e.g., phage MS2)^17^. Here, we focus on T4 bacteriophage, which belongs to *Myoviridae* family, a part of *Caudovirales* order, which comprises the vast majority of all known bacteriophages (above 95%)^18^. *Caudovirales* share a typical structure design, i.e., genetic information (double-stranded DNA) is stored in an icosahedral capsid, to which a spike-tail with fibers is attached.

One of the considered reasons for the influence of plastics on biological samples was polymer additives leaking from the container to the samples. In the polymer industry, additives are ubiquitously used in stabilizing and modifying end-product properties^19^. Almost all of the additives are not chemically bound to the polymer but instead form a solid mixture, from which compounds can be released in potentially active form^19^. Some types of additives, *e.g.,* plasticizers, slip agents, or biocides, are potentially toxic^20^. Types and used concentrations of the compounds are hidden from public view by the companies’ business confidentiality, and they vary depending on the manufacturer of plastic. However, numerous reports show the influence of leachable on biological samples, such as enzymes or eukaryotic cells. McDonald et al. proved that biocide called di(2-hydroxyethyl)methyldodecylammonium and slip agent called 9-octadecenamide were washed away from plastic and inhibited the activity of human monoamine oxidase-B^21^. Lee et al. noticed a harmful effect on hippocampal neurons culture caused by unidentified leachables from medical-grade syringes and syringe filters^22^. It was also reported that fatty acids amides (e.g., stereamide, erucamide, oleamide) were leaking from food containers into the water and olive oil^23^. The presence of bisphenol A (common plasticizer of polycarbonates and epoxy resins used in packaging) was found in body fluids in most populations of developed countries^24^. A recent study reported the presence of leachables in a vaccine^25^.

The second mechanism influencing biological samples is the adsorption of biomolecules on plastic surfaces^26^. The rate of adsorption depends on the type of the protein, material of the surface, and physicochemical conditions (such as temperature, solvent, ionic strength, etc.). Proteins interact with the surface in many ways, from which the most important are electrostatic and hydrophobic effects^27^. When deposited at the hydrophobic surfaces, such as most plastics, proteins slightly change their conformation to expose hydrophobic patches. These hydrophobic patches interact strongly with the hydrophobic surface, while more hydrophilic regions of the molecule are exposed to bulk, which reduces the system's overall energy. These hydrophobic interactions might be the driving force explaining the adsorption effect, as it was proved in the case of adsorption of collagen on polystyrene surface^28^. Krisdhasima et al. showed that in the case of some proteins (e.g., protein β-Lg), the more hydrophobic the surface, the stronger the effect of protein adsorption is^29^. The adsorption of viruses on plastic walls was mentioned previously^30,31^, but it was never thoroughly investigated and solved until now.

Plastic tubes and vials are used in life science laboratories because of ease of operation, durability, versatility, and low price. The quality and type of plastic labware are usually ignored as a factor influencing scientific results. However, as Teflon magnetic stirring bars^32^, also plasticware can cause irreproducibility of results and measurements^33^. Here, we show the effect of vendor-to-vendor and even batch-to-batch differences in properties of Eppendorf-type (standard laboratory tubes 1.5 mL or 2 mL) and Falcon-type tubes (standard laboratory tubes 50 mL), made of polypropylene, on the apparent number of active phages upon storage, mixing, and exposure to temperature. Falcon and Eppendorf tubes are registered trademarks of Corning Inc., USA, and Eppendorf AG, Germany, respectively. We do not name any vendors and producers because (1) the quality of various batches can vary even within a single vendor, and (2) the main message is to provide insight and solutions for the problem as well as to increase awareness and to improve phage-based research. We also explain the effect, provide countermeasures, and the method of verification of already possessed plastic consumables allowing to conduct of phage-related research with higher reproducibility of the results.

## Results

We tested nine polypropylene Eppendorf-type tubes denoted as E1–E9 and five polypropylene Falcon-type tubes denoted as F1–F5. All tested tubes came from different suppliers. We also compared Eppendorf-type tubes from the same vendor but from different batches (one purchased in 2018, second in 2020, see Figure S1 in Supporting Information). Glass vials were used as controls. Presented data were collected for T4 phages if not stated otherwise.

### Decrease in the number of active phages in polypropylene containers

**Figure 1:**
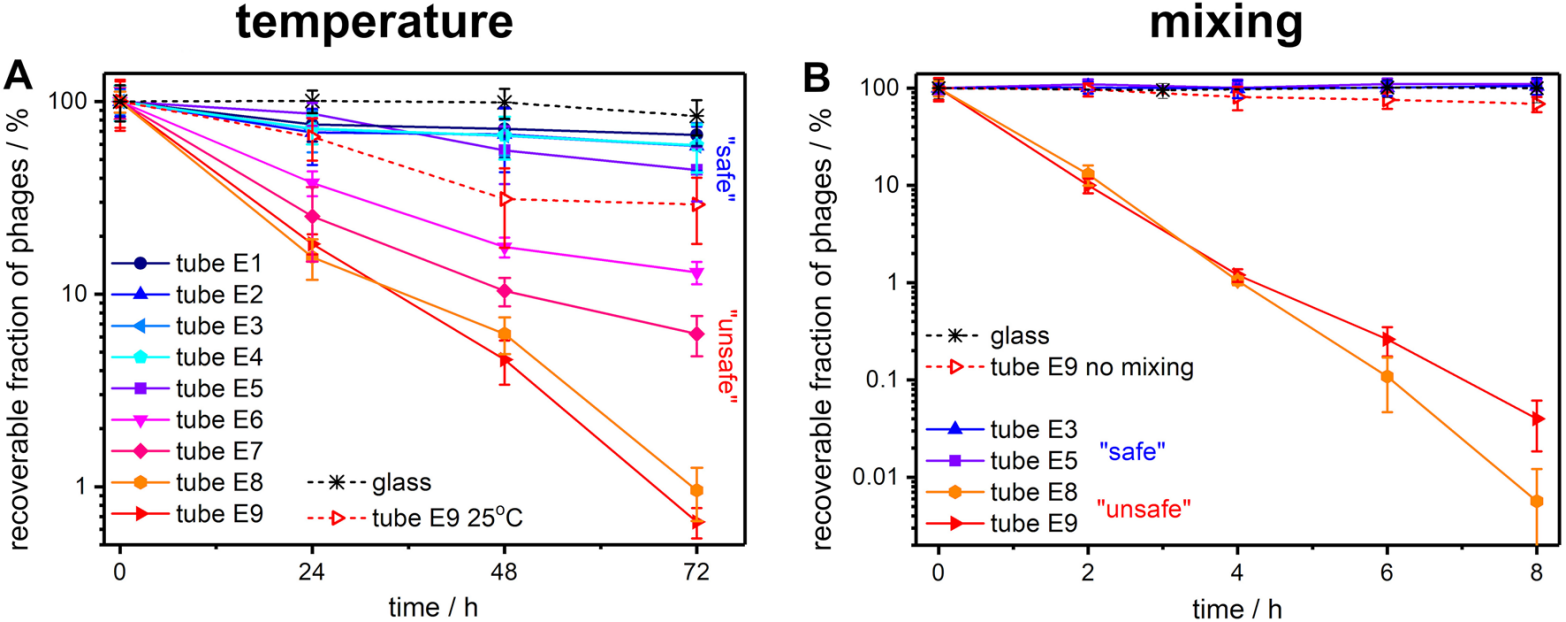
Effects of mixing (at 800 rpm) and elevated temperature (at 50 °C) on the number of active T4 phages in polypropylene Eppendorf-type tubes from various vendors.

We found that the decrease of concentration of T4 phages incubated at 50 °C for 72 h might vary from around 30% (for E5), which is typical thermal deactivation of phages in these conditions, to more than 99% (for E9) depending only on used Eppendorf-type tube (Fig. 1A). All tested Falcon-type tubes showed pronounced deactivation of T4 phages at 50 °C, similar to the worst-performing Eppendorf-type tubes (Figure S2A).

Strikingly, mixing has an even larger impact. In the Eppendorf-type tubes, the effect of mixing was bimodal—depending on the vendor of the tubes, the linear decrease (four logs within 8 h) or no effect whatsoever (Fig. 1B) was observed.

More detailed information are available in Supporting Information. The phenomenon depended on the temperature and speed of mixing (Figure S3), initial concentration of phages (Figure S4), but not on the type of used phage (Figure S5).

We classified Eppendorf-type tubes E1–E5 as “safe” to work with phages, i.e., causing relatively small changes in phage counts. Oppositely, E6–E9 were classified as “unsafe” as the uncontrolled disappearance of active phages in these tubes was significant. Despite testing five various Falcon-type tubes, we could not find a single truly “safe” one. Tube F1 performed much better than other Falcon-type tubes. In the time frame of mixing experiments, i.e., up to 8 h (notice various timescales in Fig. 1B and Figure S2B), tube F1 behaved as “safe”. Therefore, in all following experiments with mixing, we considered tube F1 as “safe”. Glass was “safe” for phages in all tested conditions, and we considered measurement in the glass container as the ultimate control sample.

### Mechanisms: leaching versus adsorption of virions

The reason for the observed differences in the phage titer could be only two: (1) phages are deactivated in the suspension (in bulk), or (2) phages interact with the surface of the container. The first scenario requires some agents to be released from the containers (e.g., additives, DNAses, RNAses, or other biomolecules, contaminations). We treated all of these agents as a single factor under the named “leachables”. The second scenario is related to tube thickness, smoothness/roughness, porosity, and chemical composition of the surface. All these factors might contribute to the adsorption of virions and apparent scavenging of phages from the bulk.

First, we verified if leachables are responsible for the observed effect of a decreasing number of active phages in polypropylene containers. We considered the possibility that leachables released from “unsafe” tubes could deactivate phages, or leachables from “safe” tubes could protect phages against external factors (e.g., temperature). First, we tried to wash out the possible leachables from both “safe” and “unsafe” tubes. Such rinsed (presumably leachable free) tubes were then used to analyze changes in the number of active phages upon mixing or at 50 °C (Figure S6A, S6D in the Supporting Information). We did not found any difference between rinsed and pristine tubes. In a second approach, we washed out the “safe”/“unsafe” tube with buffer and then transferred such buffer to the “unsafe”/“safe” one, respectively. To such (1) “safe” tubes filled with buffer containing “unsafe” leachables or (2) “unsafe” tubes filled with buffer containing “safe” leachables, we added phages and again observed the number of phages upon mixing or at 50 °C (Figure S6B, S6E). In none of these cases, we succeed in transforming “safe” to “unsafe” properties or vice versa. This excluded the leachables as suspects causing the decrease of the number of active phages from our further considerations.

**Figure 2:**
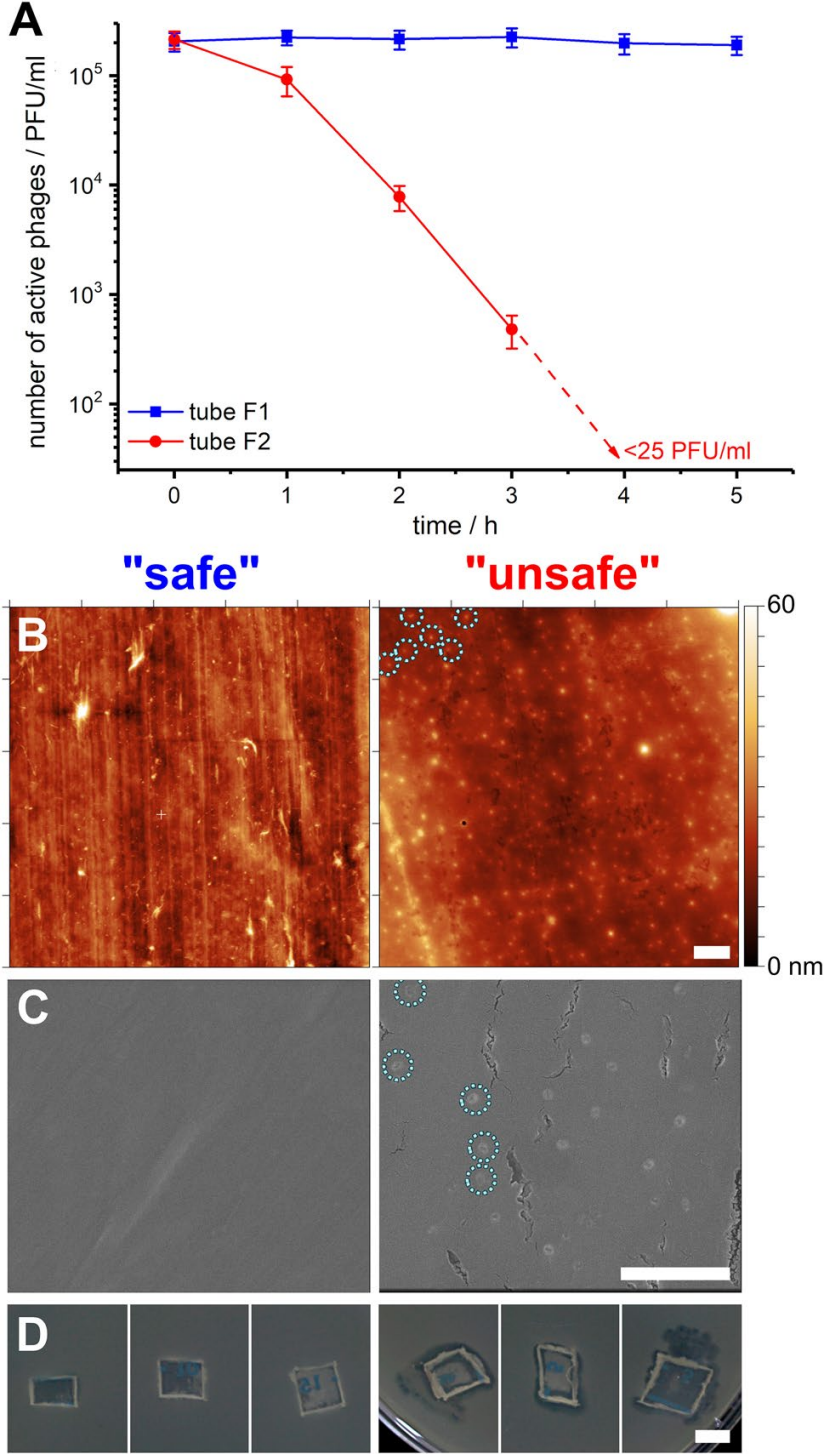
Analysis of surfaces of “unsafe” F2 and “safe” F1 tubes after incubating phages with mixing. (**A**) the number of active T4 phages is related to the surfaces of the tubes. Surfaces were analyzed using (**B**) AFM (E3 and E9) and (**C**) SEM (F1 and F2). Some virions are marked with circles. (**D**) Additionally, a piece of each plastic was placed on bacteria to check adsorbed phages' infectivity (F1 and F2). Pictures in panels (**B**–**D**) are shown with their scale bar representing 0.5 µm, 0.5 µm, and 5000 µm, respectively.

Next, we examined the adsorption of virions on the polypropylene containers' walls as a mechanism responsible for the observed effect. To verify if phages were scavenged and adsorbed at the container's surface, we used atomic force microscopy (AFM) and scanning electron microscopy (SEM). We conducted experiments in which T4 phages (10^5^–10^7^ PFU/ml) were mixed (at room temperature (RT)) in “safe” (E3 and F1) or “unsafe” (E9 and F2) tubes (Fig. 2A). After the incubation, small pieces of plastic containers were cut out, rinsed with buffer (to get rid of loosely bound virions). We found phages present at the surface of “unsafe” containers (both by means of AFM Fig. 2B, SEM Fig. 2C). There were no adsorbed phages in the case of control experiments with “safe” containers. Roughness calculated from AFM pictures is similar in the case of both “safe” and “unsafe” labware and small (in the range from 3 to 6 nm) comparing to the size of virions.

To additionally determine whether adsorbed phages retained virulence, we placed the piece of plastic, prepared according to the procedure described above, onto the agar plate containing bacteria (same as used for titration experiment) (Fig. 2D). We observed the plaques around the “unsafe” samples, whereas in the case of “safe” no effect was observed. This confirmed that at least some fraction of adsorbed phages (visualized utilizing AFM and SEM) remained active. Moreover, it appeared possible to detach virions from the walls of “unsafe” containers by adding surfactant Tween20 (cf. Section 2.4.2 and Supporting Information). Such virions remained active.

### Differences in the properties of “safe” and “unsafe” polypropylene containers

We performed X-ray photoelectron spectroscopy (XPS) measurement to evaluate differences in the chemical composition of the surface between “safe” and “unsafe” tubes (Table 1). “Safe” Eppendorf-type tubes have higher carbon and lower other elements contents than “unsafe” containers. Pure polypropylene should not contain any other detectable elements than carbon. Other elements might come from additives and post-production surface modifications.

**Table 1:** Chemical composition of the surface of “safe” and “unsafe” Eppendorf-type tubes was obtained through XPS measurements.

	% C	% O	% Ca	% Si	% S
“safe” E2	92.55	6.02	1.00	0.26	0.16
“safe” E5	91.73	8.13	0.12	0.02	0.00
“unsafe” E8	87.49	10.03	1.71	0.48	0.28
“unsafe” E9	84.87	11.89	2.09	0.79	0.34

The universal property of the surface, which originates from both its composition and morphology, is wettability. We found statistically significant differences in water wetting angles (WA) of “safe” and “unsafe” tubes. “Safe” tubes appeared less hydrophobic (or even hydrophilic) (WA of around 90°) comparing to “unsafe” (WA of around 100° and larger).

**Figure 3:**
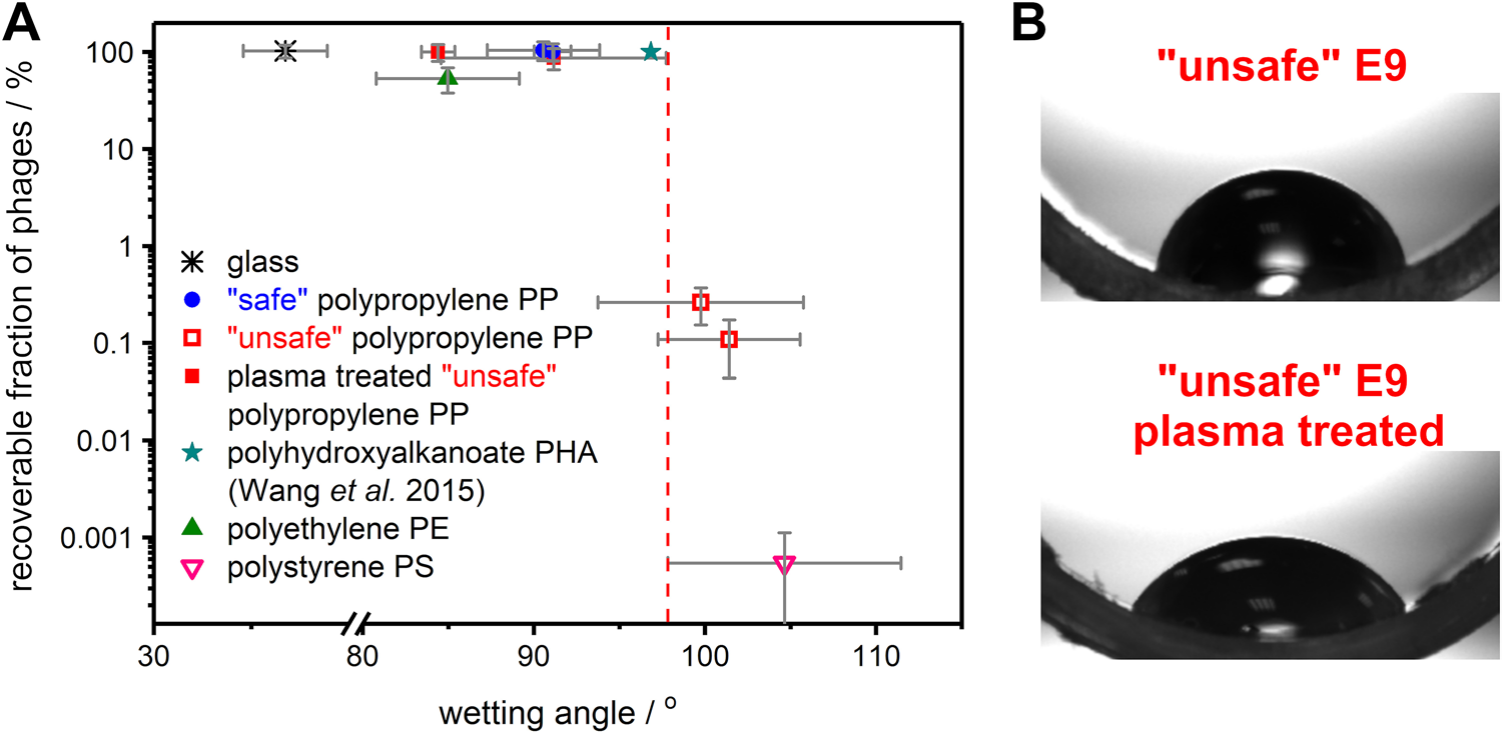
(**A**) Effect of hydrophobicity on the adsorption of phages on different types of tubes and vials. Phages T4 were mixed for 6 h, and then the loss of phages due to adsorption was estimated. (**B**) Wetting of “unsafe” E9 tube before and after plasma treatment.

We correlated wetting angles of “safe” and “unsafe” PP tubes, polystyrene (PS) tube, polyethylene (PE) tube, polyhydroxyalkanoate (PHA) surface (result extracted from Wang et al.^34^), and glass vial with the percentile decrease of the number of phages upon mixing for 6 h. We showed a clear and more general dependence between the wetting angle and phage suspension stability (Fig. 3A).

### Countermeasures

We checked if any external procedures are transforming “unsafe” to “safe” PP containers.

#### Plasma treatment of PP tubes

**Figure 4:**
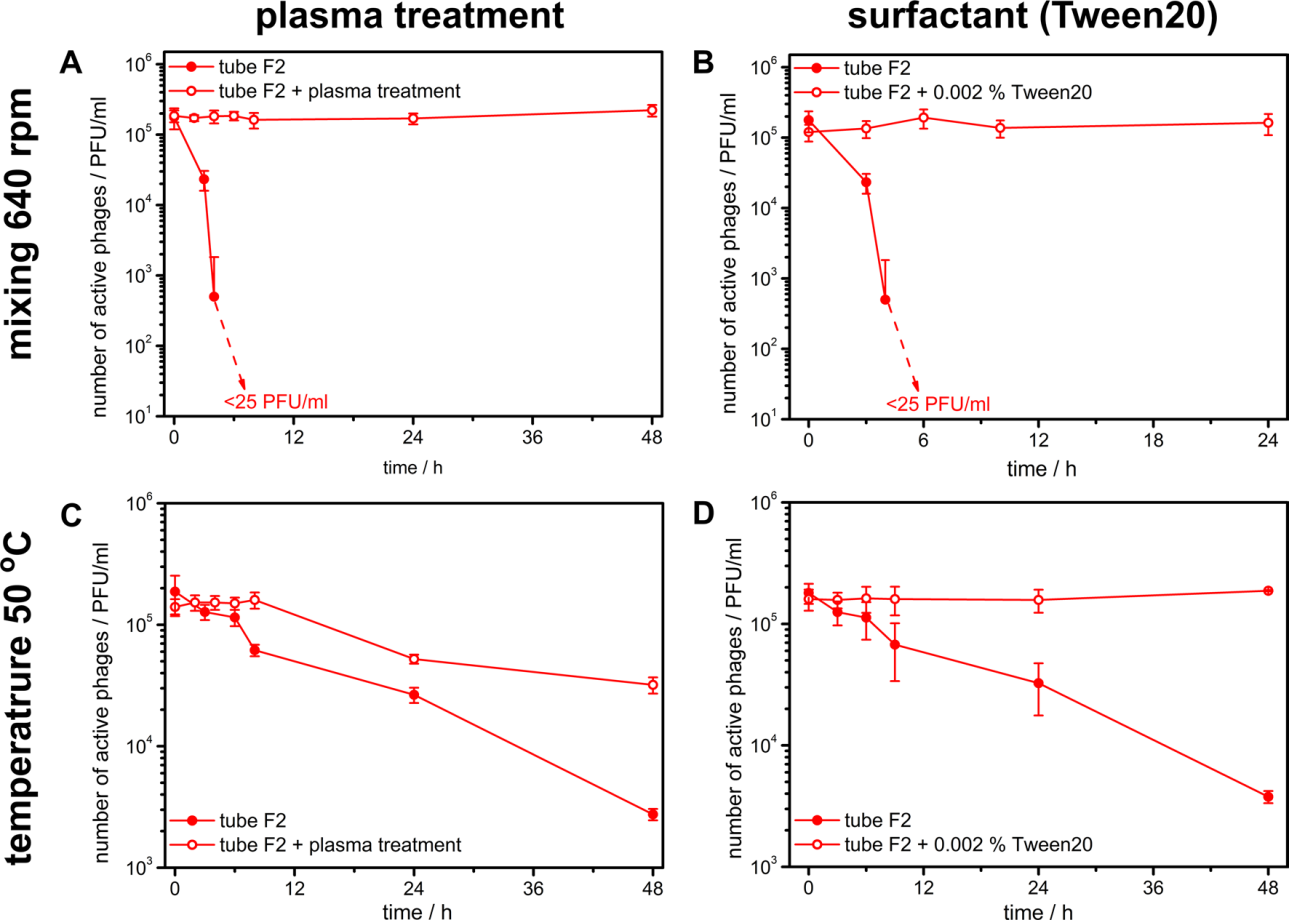
Influence of plasma treatment of PP tubes and addition of 0.002% v/v of Tween20 on the adsorption of phages on walls of the “unsafe” Falcon-type tubes. In each treatment, we measured changes in the number of active phages T4 in time in two sets of conditions: upon mixing and at temperature 50 °C.

We treated “unsafe” tubes with plasma, a standard procedure to modify plastics properties (including PP)^35–38^. Plasma treatment appears effective as a countermeasure against decreasing the number of active phages in “unsafe” containers in mixing and storage at elevated temperature (50 °C). Results for Falcon-type tube F2 are shown in Fig. 4A,C, whereas for results on “unsafe” Eppendorf-type tubes, please see Figure S7A in Supporting Information.

Transformation of “unsafe” to “safe” upon plasma treatment was accompanied by changes in WA: from 99° ± 5° to 84° ± 1° and from 101° ± 4° to 91° ± 5° for E8 and E9, respectively. This clearly corresponds to crossing a threshold suggested in Fig. 3.

#### Addition of a surfactant to the phage suspensions

All of our previous experiments confirmed that the adsorption of virions on PP containers' walls is the leading cause of the disappearance of active phages from suspensions. Thus, we tested the classical method preventing adsorption of proteins on the surfaces, namely addition of surfactant Tween20 (0.002% (v/v))^39^. We analyzed the number of phages in “unsafe” tubes (F2, Falcon-type) upon mixing (Fig. 4B) and at 50 °C (Fig. 4D) (for results on Eppendorf-type tubes see Figure S7B in Supporting Information). We observed that the addition of Tween20 inhibited the decrease and resulted in a stable number of active phages in studied conditions over prolonged time, even in “unsafe” containers. The addition of Tween20 did not have any observable effect on the phages in glass vials, thus proving this protocol's feasibility.

We observed that the addition of Tween20 results in the reappearance of active phages pre-adsorbed at the walls of plastic containers. First, we mixed T4 phages in “unsafe” tube F2. Upon 5 h of mixing, the phage titer was below the limit of detection. We continued to mix the suspensions. Tween20 was added 24 h after the start of the experiment. Phages reappeared after further 48 h, and the titer was increasing continuously until the end of the experiment (Figure S8 in Supporting Information).

## Discussion

We found that PP tubes influence the number of active phages in the phage suspension upon standard laboratory operations, e.g., mixing, heating, or upon prolonged storage. We observed that the phage titer might vary by up to 5 logs depending solely on the used tube (Fig. 1 and Figure S2). We discovered that the phage adsorption on PP occurred in both T4 and MS2, despite differences in the phages' physical structure (see Figure S5). These two phages differ not only in structure but also in electrostatic properties: T4 phage is a dipole (depending on the conformation of the fibers, the measured values of the permanent dipole moment of T4 bacteriophages varies from 20,000 to 200,000 D^40–43^) with a zeta potential of around − 26 mV^44^, whereas MS2 is negatively charged with a zeta potential of around − 40 mV^45^ (both measured in pH of around 7). Therefore, we showed that PP tubes' importance is much more general and should be considered during work with any phages.

We tested fourteen tubes, all made from polypropylene (nine Eppendorf-type and five Falcon-type) from different suppliers and different producers (details are provided in the “Materials and methods” section), which influence on the titer of phage suspensions differs by orders of magnitude. We divided studied polypropylene tubes into two groups: “safe” (E1, E2, E3, E4, E5, and F1) and “unsafe” (E6, E7, E8, E9 and F2, F3, F4, F5). It is worth noting that F1 was “safe” only against mixing and only in relatively short timescale experiments.

In all experiments, we found that glass vials were always “safe”. This confirmed that the stress caused by an external stressor (e.g., shear stress exerted upon mixing, deactivation of phages in the elevated temperatures) is not the main factor contributing to the disappearance of active phages from the solution.

We examined differences in heat transport due to variations of thickness of walls of PP tubes. Simulations showed that even in the case of walls of thickness varying by ± 90% comparing to the average value measured by us (0.5 mm), the time of reaching thermal equilibrium is below three minutes (see Figure S10 in Supporting Information). This is much shorter comparing to the time span of the experiments and did not affect experiments conducted at elevated temperatures.

We excluded leachables as the cause of the observed decrease of the phage titer based on the experiments in which we extensively rinsed the tubes with buffer. Rinsing did not change the “safe”/”unsafe” character of tested tubes, and the buffer used for rinsing, in which leachables should be present, did not affect the phages (cf. Figure S6 in Supporting Information).

Next, we showed that adsorption plays an essential role in the observed decrease in the number of active phages in plastic containers. Several observations support this. First, we analyzed the rate of transport of phages toward the container wall under the studied conditions. Without any “enhancement” of transport in the form of mixing or temperature, i.e., the decrease in the number of active phages is visible after months upon storage. An increase in temperature speeds up diffusion, and the effect started to be visible within days. Mixing had an even more significant impact (timescale in hours) on phage suspensions comparing to temperature, as convection provides more efficient transport of phages to the surface comparing to diffusion. We also observed that the magnitude of an external factor (mixing speed and temperature) is correlated with the observed decrease of phage titer (see Figure S3 in Supporting Information), which again is related to the improved transportation of phages towards the surface of the tube.

Prolonged experiments, simulating storage conditions (room temperature and no mixing), showed that the decrease of phage number reaches a plateau (Figure S4A). This is characteristic for monolayer adsorption, as, at some point, all of the adsorption centers are occupied, and no more phages can attach to the surface. Formation of the monolayer is a case for the interaction of most types of phages (excluding filamentous phages) with surfaces^46,47^.

We also found a threshold of the initial concentration of phages above which phage suspension remains stable even in “unsafe” tubes (in storage, mixing, and temperature experiments, see Figure S4). This finding again supports the adsorption of phages at the walls of PP labware. When the initial concentration of phages was high enough, loss of phages due to the adsorption was orders of magnitude smaller (due to a limited number of adsorption spots) than the initial number of phages and had a negligible effect on the overall amount of phages in the suspension. However, when the number of phages in the solution was smaller or comparable to the number of spots available for adsorption, eventually, all phages might be scavenged from the bulk. This observation is opposed to the proteins' case, where decreased concentration is suggested to slow down adsorption kinetics^48^.

SEM and AFM confirmed the presence of adsorbed phages at the surface of PP tubes. AFM pictures confirmed that after the experiments, “unsafe” tubes were covered with layer featuring objects having dozens of nm. Simultaneously, such features were not visible in the case of pristine tubes and “safe” tubes after experiments. Viral capsids adsorbed at the surface of “unsafe” tubes were clearly distinguishable in SEM pictures. Measured sizes were in the range of around 90 nm in diameter, which is precisely the size of the capsid of the T4 phage^49,50^. We additionally checked the infectivity of phages present at the surface. After each experiment, pieces of used PP tubes were washed with fresh buffer TM and incubated on a host bacteria layer (*E. coli*). In the case of “unsafe” tubes, plaques were visible, which directly proves that adsorbed phages are active and can infect and kill bacteria. No plaques were visible in the case of pristine and “safe” tubes. We were also successful in releasing phages from the surface by adding Tween20—active phages reappeared in the suspension upon adding a surfactant (see Figure S8 in Supporting Information).

We were able to correlate “safe” and “unsafe” with other measurable properties of PP tubes. From XPS measurement, it appeared that “safe” labware is produced with smaller amounts of additives, and its composition resembles more pure PP comparing to its “unsafe” counterparts. We hypothesize that the “safe” labware was plasma treated during production, whereas “unsafe” labware depends more on additives (higher content of elements, *e.g.,* sulfur, revealed by XPS) to gain desired properties. We also found a statistically meaningful difference in wetting angles between these two groups of tubes, with “safe” being less hydrophobic (WA of around 90°) comparing to “unsafe” (WA of around 100°). Results obtained by us need to be compared with these reported by Wang et al.^34^, who studied immobilization of T4 phages on polyhydroxyalkanoate (PHA) surfaces. The native surface of PHA appeared “safe”, i.e., almost no adsorption of phages was observed. The wetting angle of such surface was 96.83°, i.e., close to the threshold found by us. This agreement is contradicted by the opposite effect of plasma treatment of PHA, i.e., increased adsorption of phages with decrease WA. The wetting angle of PHA decreased from 96.83° to 14.96° upon plasma treatment. The Authors explained such dramatic change as an effect of oxygen insertion into the polymer backbone between carbons. It seems likely that this also resulted in the fragmentation of polymer chains; as a result polymer started to swell in the presence of water. Provided XPS results support this. Swelling led to an increased amount of virions both on and in the polymer.

Knowing the mechanism of the decrease in the number of active phages, we aimed to provide guides for dealing with this problem. Uncontrolled adsorption of phages and their subsequent disappearance from the solution can cause severe errors and unrepeatable results. This is extremely important for phage therapies. In the vast majority of cases, the number of active phages applied to the patient does not exceed 10^7^ PFU/ml^6,10^, i.e., it is below the “safe” threshold, and the effect might be significant.

There are three main hints coming from our studies for researchers working with phages. Firstly, we aimed to propose a simple measurement that distinguishes between “safe” and “unsafe” PP labware. This is especially important when new batches of the consumables are being used, as even the same vendor might supply tubes originating from different producers (see Figure S1 in Supporting Information). We found that the wetting angle is a useful metric for quick assessment of PP labware quality. Across various materials (polyethylene, polypropylene, polystyrene, glass), the threshold wetting angle was around 95° to 97° (“safe” is below the threshold).

Secondly, we did not find any means to transform “safe” tubes into “unsafe”. This includes autoclaving—a widely spread method of sterilization of PP labware. Even double autoclaving of PP tubes does not have any effect on “safe” and “unsafe” vials (see Figure S9 in Supporting Information). This is an important issue, as reports are showing that the sterilization conditions (e.g., dry heat, autoclave) lead to the increase of molecules binding to the surface^51^. We observed that this is not a case in phage-related research, and “safe” tubes can be autoclaved without risk.

Thirdly, we found the means to work with “unsafe” PP labware when there is such a need. Several approaches were reported to reduce adsorption, such as the addition of surfactants (*e.g.,* Tween20), high salt concentration^52^, the addition of bovine serum albumin^53^, coating of tubes with polyethylene glycol or siliconizing agents^54^, and designing a proper solvent system^55^. No methods were reported to work similarly on bacteriophages. In our first approach, we increased the hydrophilicity of the PP tubes utilizing plasma treatment^56^. We measured the number of active phages in plasma-treated “unsafe” tubes upon mixing 640 rpm (Fig. 4A) and incubated at 50 °C (Fig. 4C). We observed that in both cases, change of hydrophilicity of PP tubes (below the WA threshold, see Fig. 3) resulted in a complete transformation from “unsafe” to “safe”, which manifested as full suppression of adsorption of phages and similar stability of phage suspension as in glass vials (*i.e.,* corresponding to only thermal deactivation of phages). The second tested method was based on the addition of surfactant Tween20, which is a common way to stop the adsorption of proteins^52^. We added 0.002% of Tween20 (*i.e.* above CMC concentration, which is inherent for given surfactant) to the solution of phages and, as usual, analyzed the change of number of active phages in “unsafe” tubes upon mixing (Fig. 4B) and incubated at 50 °C (Fig. 4D). Addition of surfactant stopped the adsorption of phages and the results were similar as in glass vials.

The explanation of why phages tend to adsorb on more hydrophobic surfaces is based on the thermodynamic analysis of interactions in the system. The energy of interactions between water and the hydrophobic surface is high. It was proved that proteins could slightly change the conformation to expose hydrophobic domains, which interact strongly with the hydrophobic surface^28^. This reduces the system's overall energy as upon adsorption, hydrophilic parts of biomolecules are in contact with water. Such explanation is in agreement with the observed WA threshold (WA of “safe” PP containers below around 95°) and with experimentally validated countermeasures, i.e., the addition of Tween20 (which is better surfactant than virions) and plasma treatment (direct decrease of WA).

## Conclusions

We wish to underline that we do not correlate the name of vendors with the obtained results for three important reasons: (1) a single vendor might provide tubes from various producers without even notifying the customer; (2) the products from a single vendor might vary depending on the geographical region of the customer; (3) we found batch-to-batch variations in the case of a single vendor operating in a single geographical region, and thus we do not want to stigmatize any supplier (cf. Figure S1 in Supporting Information); (4) researchers purchase consumables from local vendors, and it is hard to predict that these vendors provide goods from the same producers as tested by us. We provide a simple method (based on measurements of wetting angle) to determine if purchased polypropylene tubes are “safe” or “unsafe”. We also provide methods for the transformation of “unsafe” to “safe” tubes.

To conclude, we tested fourteen polypropylene tubes from different suppliers and manufacturers. We showed that different types of PP containers could decrease the number of phages in solution due to the adsorption of virions on the plastic surface of WA above the threshold value. Depending on the used PP tube, the effect can vary from none (“safe” tubes) to 5 log decrease (“unsafe” tubes) in the number of phages. We showed that the observed loss of phages is caused by adsorption on the walls of PP tubes. Numerous observations confirm this hypothesis: (1) above a given initial concentration of phages, the effect disappears (when the number of adsorption centers is much lower comparing to initial phage titer), (2) decrease of phages reaches the plateau in long-time experiments (when the monolayer is formed) and (3) infective phages are present at (and might be released from) the surface of “unsafe” tubes, even when there are no detectable active virions in bulk suspension. The rate of adsorption is increased when the transport of virions towards the walls of the container is enhanced by typical laboratory procedures, e.g., increasing temperature and/or mixing.

We discovered that the adsorption of virions is governed by variability in the wettability of the PP containers. The effect was visible in polypropylene tubes from various vendors, which wetting angle varied from around 90° for “safe” to around 100° for “unsafe” containers. This threshold might be used as a benchmark for predicting the usefulness of already purchased consumables for phage research. We also established two methods to fix “unsafe” labware: (1) application of plasma treatment to PP tubes to increase hydrophilicity and (2) addition of 0.002% of Tween20 to chemically “block” the surface.

We found threshold values of concentration, above which phage suspensions remained stable against tested conditions. The threshold for T4 phages is above 10^10^ PFU/ml in the case of storage experiments, whereas 5 × 10^8^ PFU/ml was enough for suspension to remain stable upon mixing. These values are in the range of concentration used in the number of important trails utilizing phages as medical agents. The number of active phages applied to the patients is often in the range of around 10^7^ phages/ml or lower^6,10–12^. We are sure that the provided data and proposed solutions will improve all phage-based projects and contribute to the higher reproducibility of results and faster development of phage-based technologies.

Our findings are of crucial importance not only for phage-related studies. Lack of reproducibility of measurements is becoming the most significant threat to the scientific community^57^. It appeared that overlooked details, like the quality and type of used plastic labware, are essential factors contributing to the reproducibility problem. Scientists might not even be aware that PP tubes vendor might provide seemingly the same product differing significantly from batch to batch. Sometimes this can lead to false conclusions^58,59^. Or, as in the case of phages—it can prohibit the development of essential technologies due to low efficiency caused solely by improper containers.

## Materials and methods

All experiments were performed in three biological replicates (except this presented in Figure S4A (on prolonged storage), which was completed in a single biological replicate). Within each technical replicate, eight to twenty-four droplets (individual data points) were taken into account to establish phage titer for a given sample for a given time.

### Chemicals

LB-agar consisted of 15 g/L of agar, 10 g/L of NaCl, 10 g/L of tryptone, and 5 g/L of yeast extract. LB-medium had the same composition except for the lack of 15 g/L of agar. LB-agar and LB-medium were obtained in the form of instant mixes from Carl Roth (Germany). We prepared TM buffer using 10 mM Tris base, 5 μM CaCl_2_, 10 mM MgSO_4_, and distilled water. pH of the buffer was set to 7.4 using HCl (7% solution). All chemicals were purchased from Sigma Aldrich (USA). All solutions were sterilized by autoclaving (121 °C) before use.

### Tubes and vials

Examined 50 ml polypropylene Falcon-type tubes were purchased from Greiner Bio-One (Austria, Lot: E14123G4), Profilab (Poland), Carl Roth (Germany, Lot: 715CD-715C-2021-04), and SPL Life Science (Korea).

Nine types of Eppendorf-type tubes were tested. We purchased tubes from different suppliers (*e.g.,* Bionovo, Profilab, Linegal Chemicals), both colorless and colorful (black, blue), of volumes 1.5 ml and 2 ml.

Polyethylene (10 ml, LDPE) and polystyrene (4 ml) tubes were provided by Linegal Chemicals (Poland).

### Bacteriophages

To prepare T4 and MS2 phage lysate, early logarithmic cultures of *Escherichia coli* BL21 and C3000 strains, respectively, were infected. When the lysis was finished, the precipitation of phages by polyethylene glycol was conducted. The next step was centrifugation of precipitate and then re-suspension in TM buffer by using 1 M NaCl. After the mentioned procedures, T4 phages were purified by ultracentrifugation in step CsCl gradient (Becman Optima XL70 ultracentrifuge, Ti50 rotor). A cluster formed by phages after ultracentrifugation was picked by syringe aspiration. Both T4 and MS2 phages were then dialyzed against TM buffer. When dialysis was completed, 0.2 μg/ml of DNAse was added to digest DNA acquitted from phage capsids during the procedure. MS2 was filtered in order to purify as they are much smaller compared to T4.

### Bacteria

The bacteria cultures were prepared according to the standard protocol. First, a single colony from the *E. coli* (strain BL21 or C3000) stock plate was transferred to 10 ml of LB medium. The sample was incubated overnight at 37 °C in the shaker (Orbital Shaker-Incubator ES-20, 200 rpm). After 12 h the sample of *E. coli* BL21 was refreshed by adding 2.5 ml of overnight culture to 7.5 ml of LB medium and incubated in 37 °C to obtain appropriate OD_600._

### Analysis of the number of active phages in the solution

In order to determine the number of active bacteriophages, the droplet method (plaque counting) was used for tested samples. The suspension containing 4 ml of complete LB medium and 0.5% agar, mixed with 200 μl suspension of refreshed *E. coli* bacteria (strain BL21 for phage T4 and strain C3000 for phage MS2), was poured evenly onto previously prepared Petri dishes with LB-agar (LB medium and 1.5% agar). After agar solidification, at least 8 droplets of 5 µl appropriately diluted samples of bacteriophages were dropped onto each plate. When the droplets dried, the plates were placed at 37 °C and incubated for 24 h. After this time, the plaques were counted, and the concentration of the samples was calculated.

We understand that above some value, the calculation of phage titer might not be linear (e.g., due to the presence of the aggregates^60^). We compared the standard protocol and protocol with an additional step of dilution of samples of expected titer above 10^9^ PFU/ml and allowing them to equilibrate. No statistical difference was found in the studied concentration range.

### Verification of the presence of active phages on a plastic surface

To check if the phages are active and able to adsorb on the plastic surface, the following test was performed. A phage solution was added to the plastic container and was stirred for five hours until the phage concentration in the tube was zero in the solution. Then a small piece of plastic was cut out and washed with TM buffer to remove unbound phages. The piece of plastic was put on solid agar (inner surface facing up), and then it was flooded with top agar with bacteria (LB medium and 0.5% agar, mixed with 200 μl suspension of refreshed *E. coli* bacteria). As a control, we used a cropped out plastic from a plastic container in which no phages were mixed.

### Incubation upon mixing or at elevated temperatures

10 ml of T4 phage suspension of concentration 2 × 10^5^ PFU/ml in TM buffer were tested in 50 ml polypropylene Falcon-type tubes. Tubes were installed on IKA KS130 Control Shaker (Germany) and stirred for 48 h at 200 rpm, 400 rpm, or 640 rpm at room temperature (RT). As a control, we used 50 ml glass vial.

1.5 ml Eppendorf-type tubes were mixed for 8 h using Thermo-Shaker TS-100C (BioSan) at 800 rpm, at room temperature (RT). The volume of the samples was set to 700 μl to ensure the formation of a proper vortex. The concentration of T4 phages was 4 × 10^6^ PFU/ml.

10 ml of T4 phage suspension of concentration 2 × 10^5^ PFU/ml in TM buffer were incubated at 50 °C using temperature safety device DIN 12,880 (Binder).

1.5 ml Eppendorf-type tubes were incubated without mixing at 50 °C using Dry Block Thermostat Bio TBD-100 (BioSan). To maintain 37 °C, tubes were placed in temperature safety device DIN 12,880 (Binder).

### Removal or transfer of leachables from plastics

“Safe” (E5) and “unsafe” (E8) Eppendorf-type tubes were filled with TM buffer and heated for 24 h at 50 °C. T4 phage suspensions of the final concentration of around 4 × 10^6^ PFU/ml in TM buffer were used to test such pretreated tubes.

50 ml polypropylene Falcon-type tubes (“safe” F1 and “unsafe” F2) and 50 ml glass vial were all filled with 10 ml of TM buffer and stirred for 12 h at 640 rpm (room temperature, RT). T4 phage suspensions of the final concentration of around 2 × 10^5^ PFU/ml in TM buffer were used to test such pretreated tubes. Results are presented in Supporting Information (Figure S4).

### Autoclaving

To check the influence of autoclaving on the tubes, the following experiment was performed: the solution of T4 bacteriophages of concentration 4 × 10^6^ PFU/ml was incubated at 50 °C in (a) E5 (“safe”) tube without autoclaving, (b) E5 (“safe”) tube 1 × autoclaved and (c) E5 (“safe”) tube 2 × autoclaved, (d) E8 (“unsafe”) tube without autoclaving and (e) E8 (“unsafe”) tube 1 × autoclaved. The elevated temperature was maintained for 48 h, and the number of active phages was measured along with the experiment.

### Tween20

To 50 ml polypropylene Falcon-type tube and 50 ml glass vial (both filled with 10 ml solution of 2 × 10^5^ PFU/ml T4 phages in TM buffer), Tween20 was added to a final concentration of 0.002% v/v. As a control, the same tubes but without Tween20 were tested. Tubes were incubated at 50 °C for 24 h. The same experiment was replicated, but instead of incubation at 50 °C, samples were stirred at 640 rpm at room temperature (RT).

### Plasma treatment of PP tubes

In this study 50 ml Falcon-type and 1.5 ml Eppendorf-type tubes were treated with oxygen plasma by using Harrick Plasma Expanded Plasma Cleaner. We performed this experiment using typical conditions used for plasma cleaning. Plasma treatment was performed through Reactive Ion Etching (μEtch) in a cylindrical chamber (6 in. diam. × 6.5 in. L Pyrex chamber). Commercially available oxygen gas (99.993%, Praxair, Edmonton, Canada) was input into the chamber. Samples were treated in plasma at a floating potential for 30 s under a RF power of 30 W and an oxygen concentration of 25%, and a vacuum of 200 mTorr.

### Scanning electron microscopy and atomic force microscopy

Samples for SEM were prepared as mentioned in section “Verification of the presence of active phages on a plastic surface”. First phages were mixed (800 rpm, concentration of T4 phages 4 × 10^6^ PFU/ml, 8 h for Eppendorf-type tubes, and 640 rpm, the concentration of T4 phages 2 × 10^5^ PFU/ml, 5 h for Falcon-type tubes). Afterward, a rectangular plastic piece was cut from Eppendorf-type tubes (E5, E6) or Falcon-type tubes (F1, F2). Atomic force microscopy imaging was performed with a MultiMode Scanning Probe Microscope (Bruker, USA). For SEM analysis, plastic pieces were covered with gold using sputter (EMITECH K550X) (thickness of the gold layer: 9–11 nm). Scanning electron microscopy images were taken with the use of NovaSEM (FEI USA).

### X-ray photoelectron spectroscopy

The XPS spectra were recorded with a PHI 5000 VersaProbe (ULVAC-PHI) scanning ESCA Microprobe using monochromatic Al-Kα radiation (hν = 1486.6 eV) from an X-ray source operating at 100 μm spot size, 25 W power, and 15 kV acceleration voltage. The spectrometer was equipped with a spherical capacitor energy analyzer with multi-channel detection. Both survey and high-resolution (HR) XPS spectra were collected with the analyzer pass energy of 117.4 and 23.5 eV and the energy step size of 0.4 and 0.1 eV, respectively, from surface 250 per 250 mm. CasaXPS ver. 2.3.23. PR1.0 software was used to analyze the XPS data using sensitivity factors database from spectrometer producer Physical Electronics/ULVAC. Shirley background subtraction and peak fitting with Gaussian–Lorentzian-shaped profiles (30% Lorentzian character) was performed. For quantification, the PHI Multipak sensitivity factors and determined transmission function of the spectrometer were used.

### Measurement of wetting angle

The contact angle was measured by the sessile drop technique using the goniometer (NanoScience Instruments) and OneAttension ver. 1.0 software. The measuring liquid was deionized water, and the volume of the droplet was approx. 10 µl. Small pieces of pristine plastic tubes (3 pieces per each tube), both Falcon-type (F1, F2, F3) and Eppendorf-type (E1, E2, E3, E4, E6, E9), were cut out and then tested on the equipment. The wetting angle of E6 and E9 after plasma treatment was measured as well.

## Supplementary Information

Supplementary Information

## Data Availability

The datasets used and/or analyzed during the current study are available from the corresponding author on reasonable request.

## Abbreviations

AFM: Atomic force microscopy
PFU: Plaque-forming unit
PE: Polyethylene
PHA: Polyhydroxyalkanoate
PP: Polypropylene
PS: Polystyrene
rpm: Revolutions per minute
RT: Room temperature
SEM: Scanning electron microscopy
WA: Wetting angle
XPS: X-ray photoelectron spectroscopy

## References

[1] Sulakvelidze A, Alavidze Z, Glenn Morris J (2001). Bacteriophage therapy. Antimicrob. Agents Chemother..

[2] Summers WC (2012). The strange history of phage therapy. Bacteriophage.

[3] Ventola CL (2015). The antibiotic resistance crisis: Part 1: Causes and threats. Pharm. Ther..

[4] Ventola CL (2015). The antibiotic resistance crisis part 2: Management strategies and new agents. Pharm. Ther..

[5] Lin DM, Koskella B, Lin HC (2017). Phage therapy: An alternative to antibiotics in the age of multi-drug resistance. World J. Gastrointest. Pharmacol. Ther..

[6] Wright A, Hawkins CH, Änggård EE, Harper DR (2009). A controlled clinical trial of a therapeutic bacteriophage preparation in chronic otitis due to antibiotic-resistant *Pseudomonas aeruginosa*; A preliminary report of efficacy. Clin. Otolaryngol..

[7] Leitner L (2020). Intravesical bacteriophages for treating urinary tract infections in patients undergoing transurethral resection of the prostate: A randomised, placebo-controlled, double-blind clinical trial. Lancet Infect. Dis..

[8] Speck P, Smithyman A (2015). Safety and efficacy of phage therapy via the intravenous route. FEMS Microbiol. Lett..

[9] Aslam S (2020). Lessons learned from the first 10 consecutive cases of intravenous bacteriophage therapy to treat multidrug-resistant bacterial infections at a single center in the United States. Open Forum Infect. Dis..

[10] Jault P (2018). Efficacy and tolerability of a cocktail of bacteriophages to treat burn wounds infected by *Pseudomonas aeruginosa* (PhagoBurn): A randomised, controlled, double-blind phase 1/2 trial. Lancet Infect. Dis..

[11] LaVergne S (2018). Phage therapy for a multidrug-resistant *Acinetobacter baumannii* craniectomy site infection. Open Forum Infect. Dis..

[12] Międzyborski R (2017). Means to facilitate the overcoming of gastric juice barrier by a therapeutic staphylococcal bacteriophage A5/80. Front. Microbiol..

[13] Sulakvelidze A (2013). Using lytic bacteriophages to eliminate or significantly reduce contamination of food by foodborne bacterial pathogens. J. Sci. Food Agric..

[14] Karimi M (2016). Bacteriophages and phage-inspired nanocarriers for targeted delivery of therapeutic cargos. Adv. Drug Deliv. Rev..

[15] Richter Ł, Janczuk-Richter M, Niedziółka-Jönsson J, Paczesny J, Hołyst R (2018). Recent advances in bacteriophage-based methods for bacteria detection. Drug Discov. Today.

[16] Janczuk M, Niedziółka-Jönsson J, Szot-Karpińska K (2016). Bacteriophages in electrochemistry: A review. J. Electroanal. Chem..

[17] Jain R, Srivastava R (2009). Metabolic investigation of host/pathogen interaction using MS2-infected *Escherichia coli*. BMC Syst. Biol..

[18] Ackermann HW (2007). 5500 Phages examined in the electron microscope. Arch. Virol..

[19] Hahladakis JN, Velis CA, Weber R, Iacovidou E, Purnell P (2018). An overview of chemical additives present in plastics: Migration, release, fate and environmental impact during their use, disposal and recycling. J. Hazard. Mater..

[20] Grzeskowiak, R., Gerke, N. & Ag, E. Leachables: Minimizing the Influence of Plastic Consumables on the Laboratory Workflows. 1–6 (2015).

[21] McDonald GR (2008). Bioactive contaminants leach from disposable laboratory plasticware. Science.

[22] Lee TW, Tumanov S, Villas-Bôas SG, Montgomery JM, Birch NP (2015). Chemicals eluting from disposable plastic syringes and syringe filters alter neurite growth, axogenesis and the microtubule cytoskeleton in cultured hippocampal neurons. J. Neurochem..

[23] Cooper I, Tice PA (1995). Migration studies on fatty acid amide slip additives from plastics into food simulants. Food Addit. Contam..

[24] Vandenberg LN, Hauser R, Marcus M, Olea N, Welshons WV (2007). Human exposure to bisphenol A (BPA). Reprod. Toxicol..

[25] Zhang Y (2016). Detection and identification of Leachables in vaccine from plastic packaging materials using UPLC-QTOF MS with self-built polymer additives library. Anal. Chem..

[26] Nakanishi K, Sakiyama T, Imamura K (2001). On the adsorption of proteins on solid surfaces, a common but very complicated phenomenon. J. Biosci. Bioeng..

[27] Rabe M, Verdes D, Seeger S (2011). Understanding protein adsorption phenomena at solid surfaces. Adv. Colloid Interface Sci..

[28] Woodcock SE, Johnson WC, Chen Z (2005). Collagen adsorption and structure on polymer surfaces observed by atomic force microscopy. J. Colloid Interface Sci..

[29] Krisdhasima V, McGuire J, Sproull R (1992). Surface hydrophobic influences on β-lactoglobulin adsorption kinetics. J. Colloid Interface Sci..

[30] Moldovan R, Chapman-McQuiston E, Wu XL (2007). On kinetics of phage adsorption. Biophys. J..

[31] Butot S (2007). Attachment of enteric viruses to bottles. Appl. Environ. Microbiol..

[32] Pentsak EO, Eremin DB, Gordeev EG, Ananikov VP (2019). Phantom reactivity in organic and catalytic reactions as a consequence of microscale destruction and contamination-trapping effects of magnetic stir bars. ACS Catal..

[33] Baker M, Dolgin E (2017). Cancer reproducibility project releases first results. Nature.

[34] Wang C, Sauvageau D, Elias A (2016). Immobilization of active bacteriophages on polyhydroxyalkanoate surfaces. ACS Appl. Mater. Interfaces.

[35] Kaplan SL, Rose PW (1991). Plasma surface treatment of plastics to enhance adhesion. Int. J. Adhes. Adhes..

[36] Occhiello E, Morra M, Morini G, Garbassi F, Humphrey P (1991). Oxygen-plasma-treated polypropylene interfaces with air, water, and epoxy resins: Part I. Air and water. J. Appl. Polym. Sci..

[37] Oravcova A, Hudec I (2010). The influence of atmospheric pressure plasma treatment on surface properties of polypropylene films. Acta Chim. Slovaca.

[38] Wolf R, Sparavigna AC (2010). Role of plasma surface treatments on wetting and adhesion. Engineering.

[39] Pan H, Xia Y, Qin M, Cao Y, Wang W (2015). A simple procedure to improve the surface passivation for single molecule fluorescence studies. Phys. Biol..

[40] Greve J, Block J (1973). Transient electric birefringence of T-even bacteriophages. I. T4B in the absence of tryptophan and fiberless T4 particles. Biopolymers.

[41] Greve J, Blok J (1975). Transient electric birefringence of T-even bacteriophages. II. T4B in the Presence of tryptophan and T4D. Biopolymers.

[42] Boontje W, Greve J, Blok J (1977). Transient electric birefringence of T-even bacteriophages. III. T2L and T6 with retracted fibers compared with T4B. Biopolymers.

[43] Boontje W, Greve J, Blok J (1978). Transient electric birefringence of T-even bacteriophages. IV. T2L0 and T6 with extended tail fibers. Biopolymers.

[44] Hosseinidoust Z, Van De Ven TGM, Tufenkji N (2011). Bacterial capture efficiency and antimicrobial activity of phage-functionalized model surfaces. Langmuir.

[45] Armanious A (2016). Viruses at solid-water interfaces: A systematic assessment of interactions driving adsorption. Environ. Sci. Technol..

[46] Richter Ł (2016). Ordering of bacteriophages in the electric field: Application for bacteria detection. Sensors Actuators B Chem..

[47] Richter Ł (2017). Dense layer of bacteriophages ordered in alternating electric field and immobilized by surface chemical modification as sensing element for bacteria detection. ACS Appl. Mater. Interfaces.

[48] Vogler EA (2012). Protein adsorption in three dimensions. Biomaterials.

[49] Abuladze NK, Gingery M, Tsai J, Eiserling FA (1994). Tail length determination in bacteriophage T4. Virology.

[50] Comeau AM, Bertrand C, Letarov A, Tétart F, Krisch HM (2007). Modular architecture of the T4 phage superfamily: A conserved core genome and a plastic periphery. Virology.

[51] Kofanova OA, Mommaerts K, Betsou F (2015). Tube polypropylene: A neglected critical parameter for protein adsorption during biospecimen storage. Biopreserv. Biobank..

[52] Smith JA, Hurrell JGR, Leach SJ (1978). Elimination of nonspecific adsorption of serum proteins by Sepharose-bound antigens. Anal. Biochem..

[53] Felgner PL, Wilson JE (1976). Hexokinase binding to polypropylene test tubes. Artifactural activity losses from protein binding to disposable plastics. Anal. Biochem..

[54] Kramer KJ (1976). Purification and characterization of the carrier protein for juvenile hormone from the hemolymph of the tobacco hornworm Manduca sexta Johannson (Lepidoptera: Sphingidae). J. Biol. Chem..

[55] Suelter CH, Deluca M (1983). How to prevent losses of protein by adsorption. Anal. Biochem..

[56] Recek N (2014). Adsorption of proteins and cell adhesion to plasma treated polymer substrates. Int. J. Polym. Mater. Polym. Biomater..

[57] Baker M, Penny D (2016). Is there a reproducibility crisis?. Nature.

[58] Lewis LK, Robson MH, Vecherkina Y, Ji C, Beall GW (2010). Interference with spectrophotometric analysis of nucleic acids and proteins by leaching of chemicals from plastic tubes. Biotechniques.

[59] Grzeskowiak R, Hübler D (2018). Is that really DNA in your tube ? Comparative analysis of UV-absorbing leachables in micro-test tubes. Appl. Note No..

[60] Szermer-Olearnik B (2017). Aggregation/dispersion transitions of T4 phage triggered by environmental ion availability. J. Nanobiotechnology.

