# Supplementary material for: Adsorption of bacteriophages on polypropylene labware affects the reproducibility of phage research
Source: Sci Rep. 2021 Apr 1;11:7387. doi: 10.1038/s41598-021-86571-x (PMC8016829; doi:10.1038/s41598-021-86571-x)
Supplement: Supplementary file 1 — Supplementary Information [file 41598_2021_86571_MOESM1_ESM.docx]

Supporting Information

Adsorption of bacteriophages on polypropylene labware affects the reproducibility of phage research

Łukasz Richter,^1^ Karolina Księżarczyk,^1^ Karolina Paszkowska,^1^ Marta Janczuk‑Richter,^1^ Joanna Niedziółka-Jönsson,^1^ Jacek Gapiński,^2^ Marcin Łoś,^3,4^ Robert Hołyst,^1,*^ Jan Paczesny^1,*^

^1^Institute of Physical Chemistry of the Polish Academy of Sciences, Kasprzaka 44/52, 01-224 Warsaw, Poland

^2^Department of Molecular Biophysics, Adam Mickiewicz University in Poznań, 61-614, Poznań, Poland

^3^Department of Molecular Biology, University of Gdansk, Wita Stwosza 59, 80-308 Gdansk, Poland

^4^Phage Consultants, Partyzantów 10/18, 80-254 Gdansk, Poland





**Figure S1.** Batch-to-batch variability in tubes purchased from single vendor affects phage studies.

*Influence of intensity of mixing and temperature*

We aimed to evaluate the effect of temperature and mixing to establish when plastic labware becomes “unsafe”. We tested three temperatures (25 °C, 37 °C, and 50 °C) and three mixing speeds (200 rpm, 400 rpm, and 640 rpm), in both “safe” F1 and “unsafe” F2 tubes. At both studied elevated temperatures, the decrease of active phages was similar. The intensity of mixing strongly and gradually affected phage suspension's stability in a whole range of tested parameters.

In four out of five testes Falcon-type tubes, 5 log decrease was observed within 5 hours of mixing at 640 rpm. One of the Falcon-type tubes (F1) caused no effect on phage suspension upon 5 hours of mixing. The decrease was eventually observed also in F1 but in a much longer (dozens of hours) time frame (**Figure S2B**).


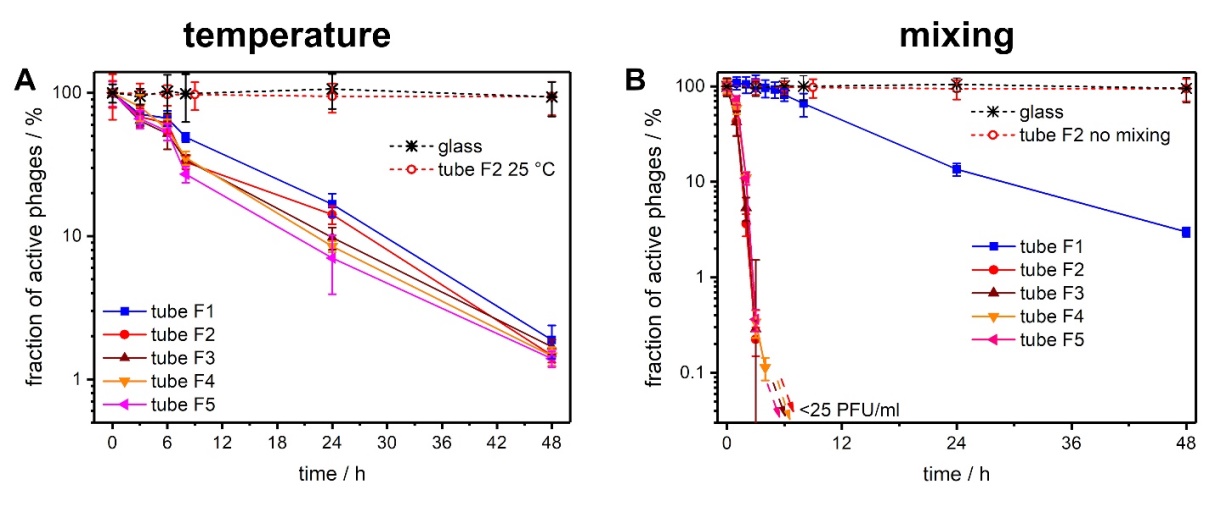


**Figure S2.** Effects of mixing and elevated temperature on the number of active T4 phages in polypropylene tubes from various vendors. The elevated temperature was set to 50 °C. Mixing was performed at 640 rpm in the case of Falcon-type tubes. Dashed arrows indicate that number of phages in the next time point decreased below the limit of detection.

**

Figure S3.** Influence of various (**A**) temperatures and (**B**) intensities of mixing on the number of active T4 phages in “safe” Falcon-like tube F1 (upper row) and “unsafe” F2 tube (bottom row). Dashed lines indicate that number of phages in the next measurement point decreased below the limit of detection (0 plagues in all 8 droplets).

**B**

**A**


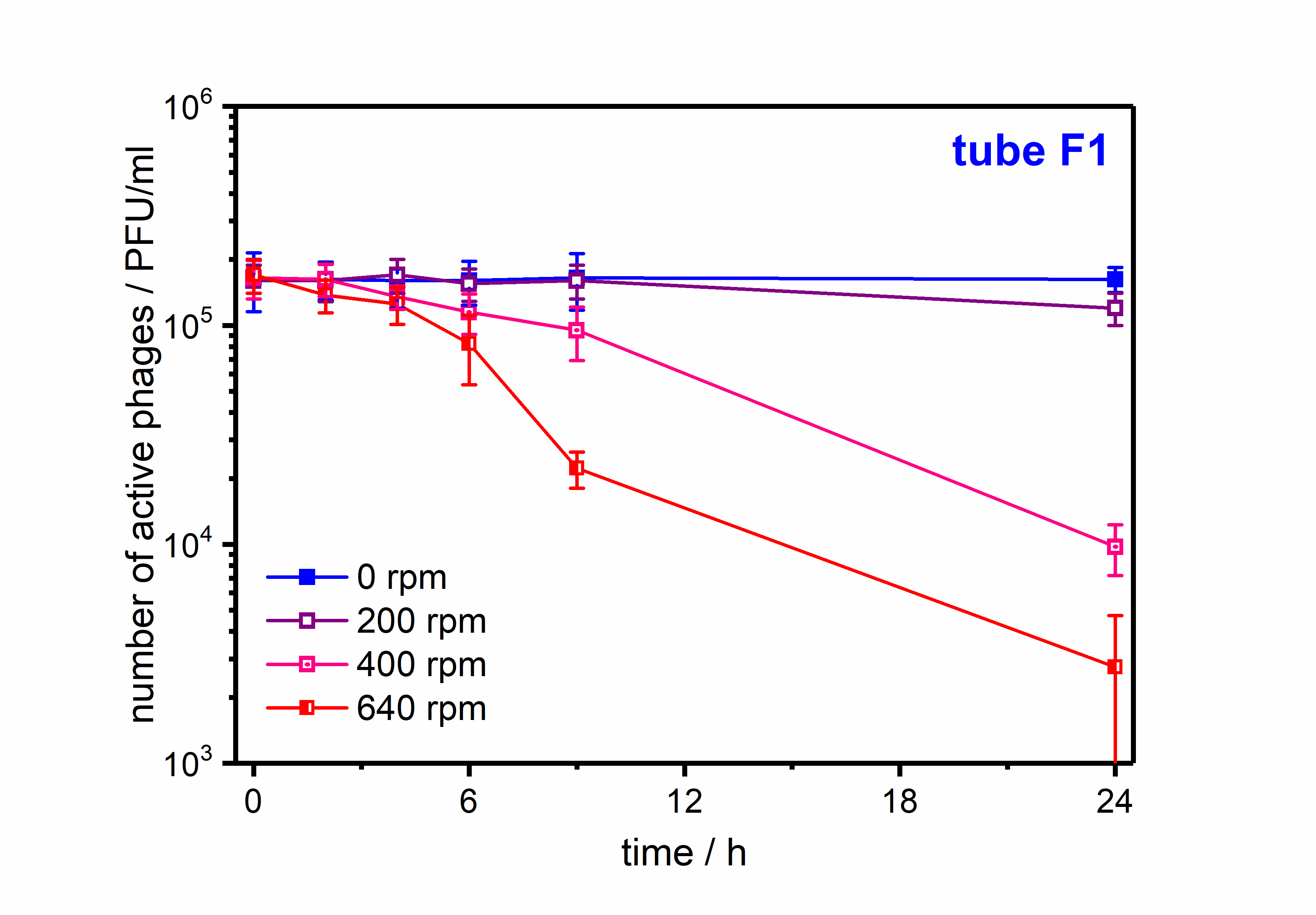

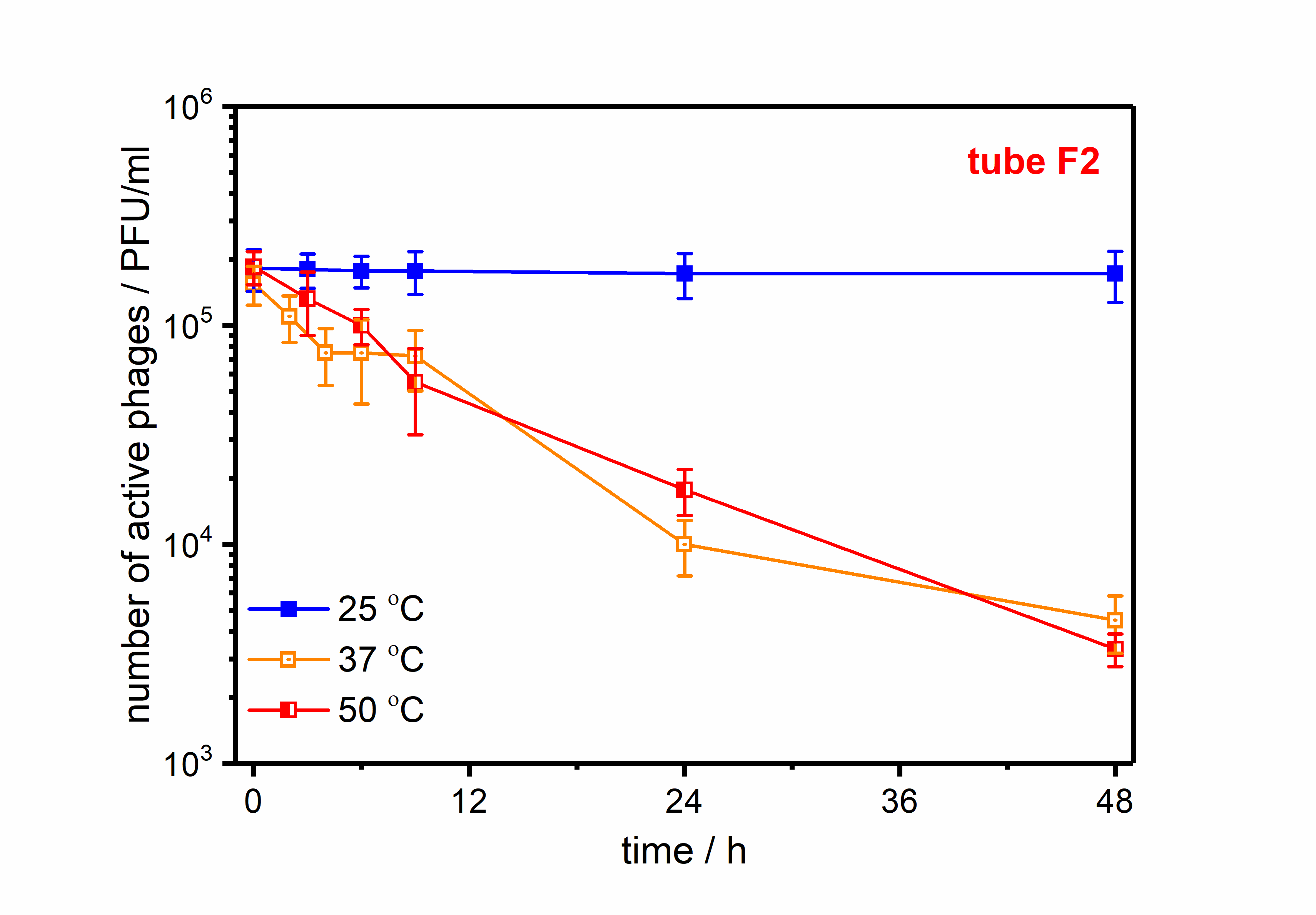

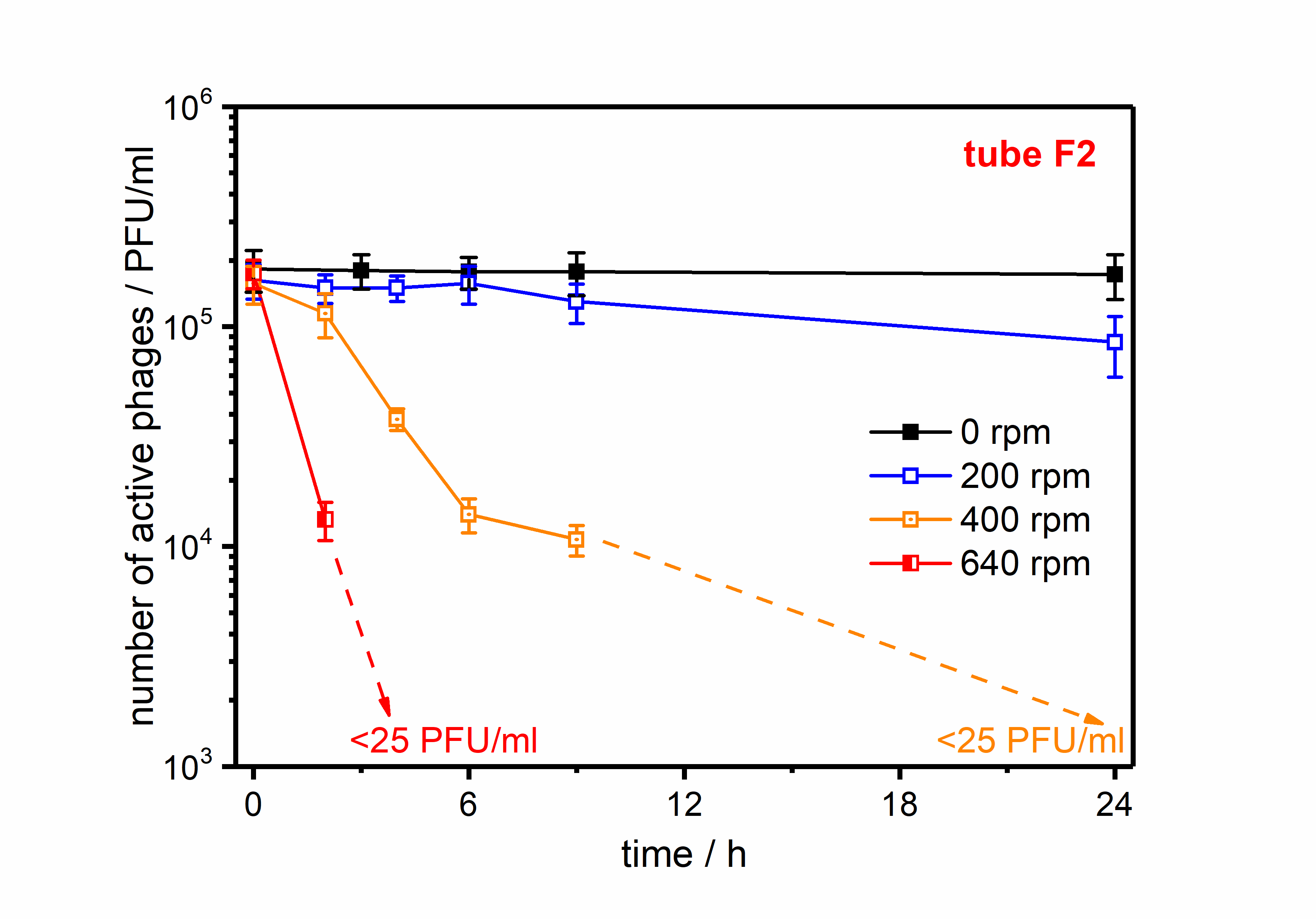


Next, we studied the influence of phage suspension's initial concentration on the effect of uncontrolled disappearance of phages upon prolonged storage, mixing, and elevated temperature. Experiments were performed in “unsafe” polypropylene tubes.

\
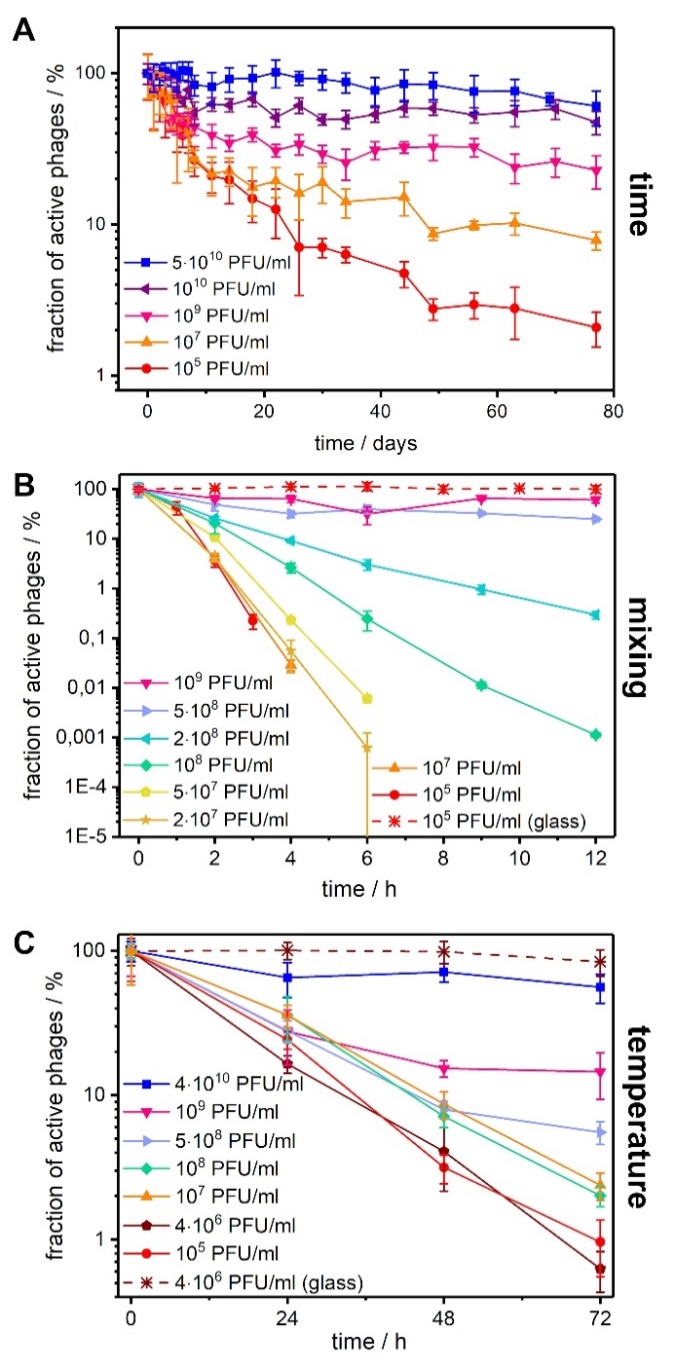


**Figure S4.** Effect of initial concentration of phages T4 on decrease of number of phages due to adsorption on plastic surface in variety of conditions: **A**) no mixing in 25 °C in tube F2; **B**) mixing 640 rpm in 25 °C in tube F2; **C**) no mixing at 50 °C in tube E9. Please note the differences in timescales and various time units. Both F2 and E9 were classified as “unsafe”.

*MS2 bacteriophage*

MS2 consists mostly of one type of coat protein and one type of maturation protein ^1^, whereas T4 consists of 5 capsid proteins and 30 other proteins ^2^.

**B**

**A**


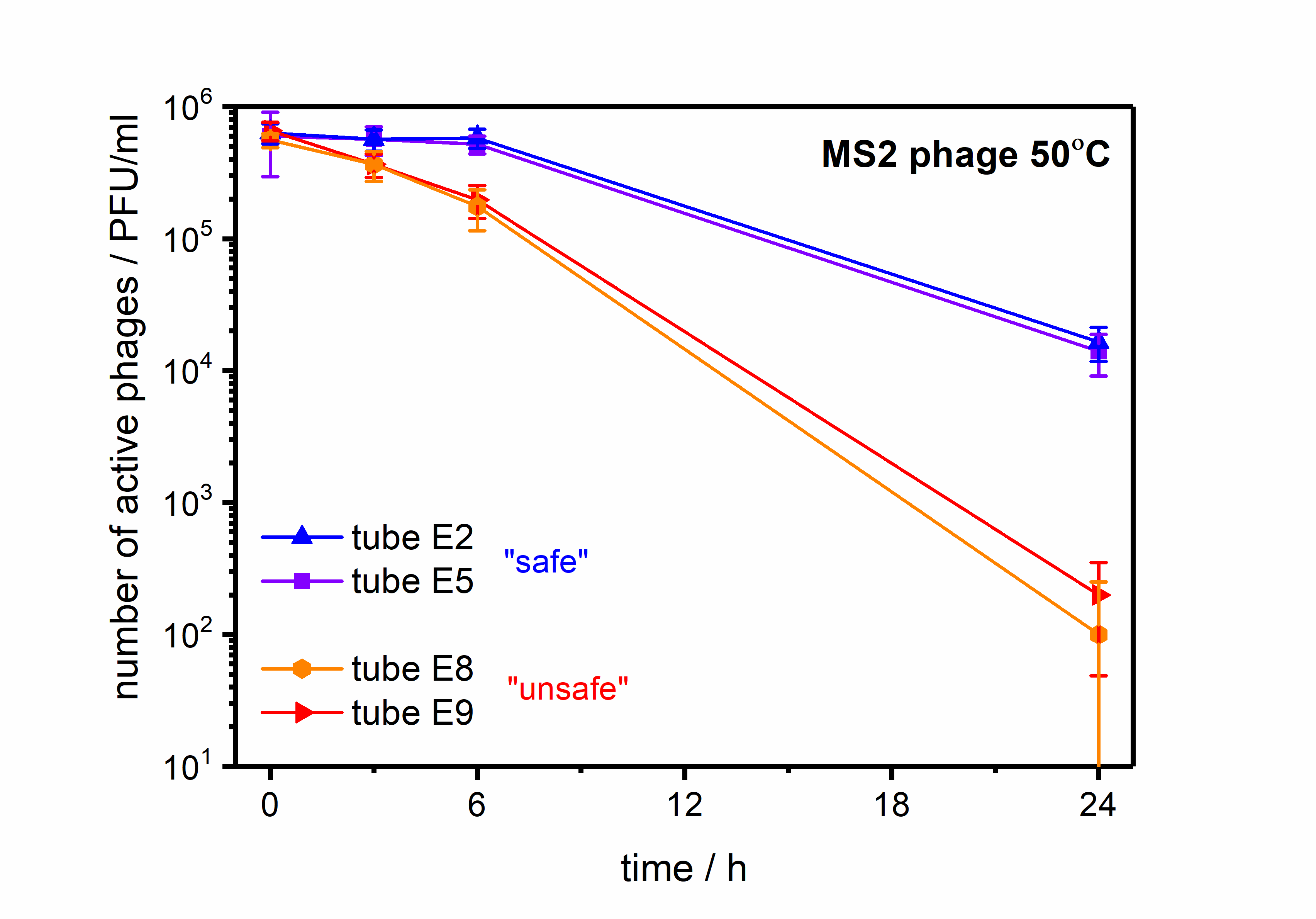

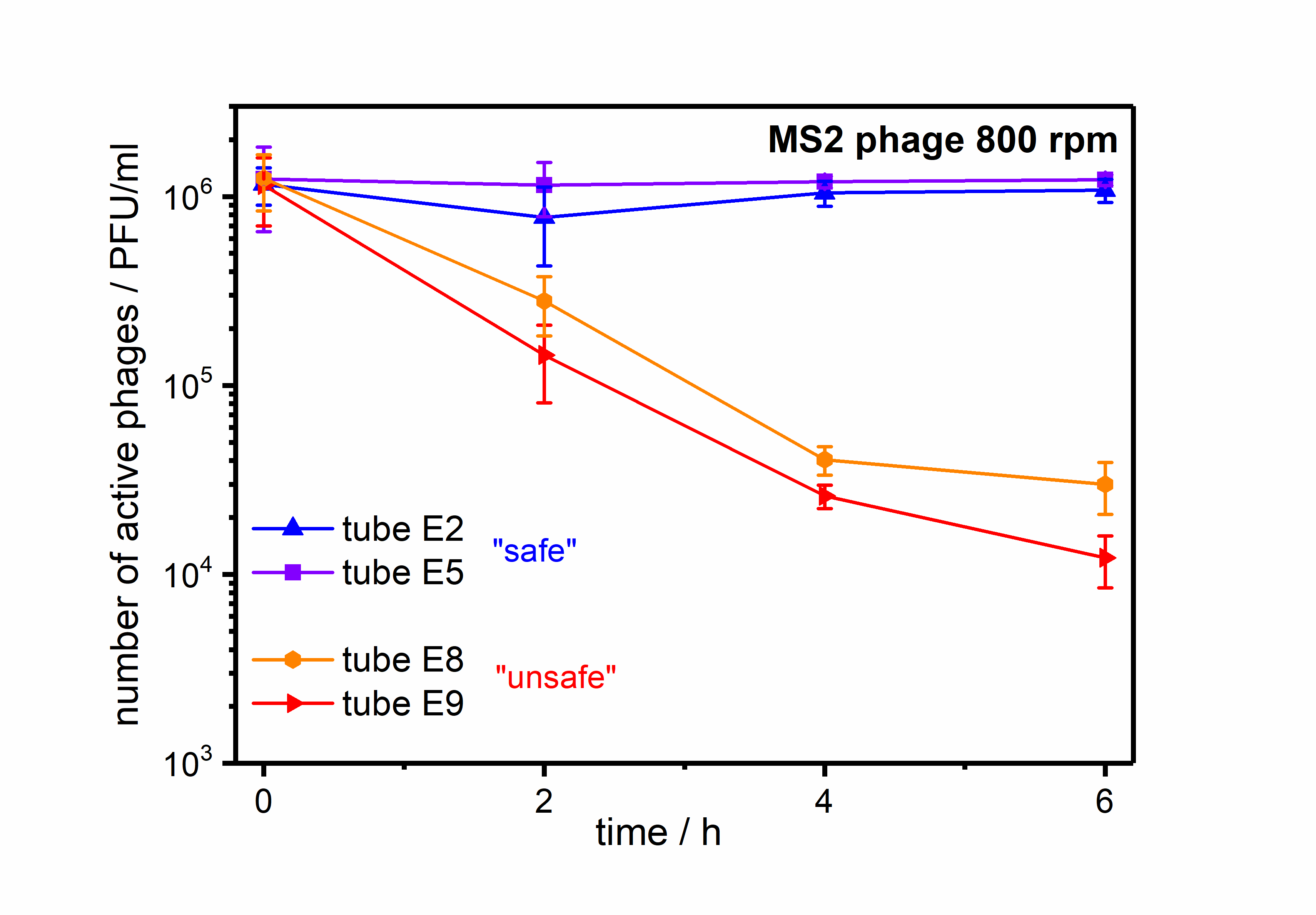


**Figure S5.** Effect of temperature (50 °C) (**A**) and mixing (800 rpm) (**B**) on the number of active MS2 bacteriophages. There is a clear difference between “safe” and “unsafe” labware in the case of both T4 and MS2 phages (*cf.* **Figure 1** and **Figure S2**).

*Effect of agents released from the containers (“leachables”)*

One of the reported causes of the influence of plastics on biological samples is the leakage of polymer additives from container to sample ^3–5^. We aimed to examine if the observed effect is solely due to adsorption, or we observed mixed effect and leachables also play an important role in decreasing the number of active phages. To investigate if any leachables influence bacteriophages in a positive or negative way, we performed a series of experiments in which we tried to remove leachables from the containers or transfer them between tubes. First, fresh TM buffer (without phages) was mixed for 12 h at 640 rpm (Falcon-type tubes; F1 (“safe”) vs. F2 (“unsafe”)) or heated to 50 °C for 7 days (Eppendorf-type tubes; E1 (“safe”) vs E9 (“unsafe”)) to wash out all the potential leachables. Such conditions were found before to cause apparent deactivation of phages. Next, two approaches were tested:

1. Washed out “unsafe” (**Figure S6A**) or “safe” (**Figure S6D**) tubes were used for “standard” experiments in which deactivation of T4 phages upon mixing or at elevated temperature was evaluated. There was no difference between washed out and pristine tubes, which proved that it is impossible to wash out all leachables to make “unsafe” tube “safe” and vice versa.
2. Buffer mixed for 12 h or heated for one week (possibly containing leachables) in “safe” (**Figure S6B**) or “unsafe” (**Figure S6E**) tubes was afterward transferred to fresh (pristine) “unsafe” or “safe” tube, respectively. Next, we added a small amount of highly concentrated T4 phage suspension and evaluated the deactivation of phages upon mixing or at elevated temperature. We proved that it is impossible to transfer ”safe” or “unsafe” properties with buffer, and thus leachables are not involved in the observed phenomenon.


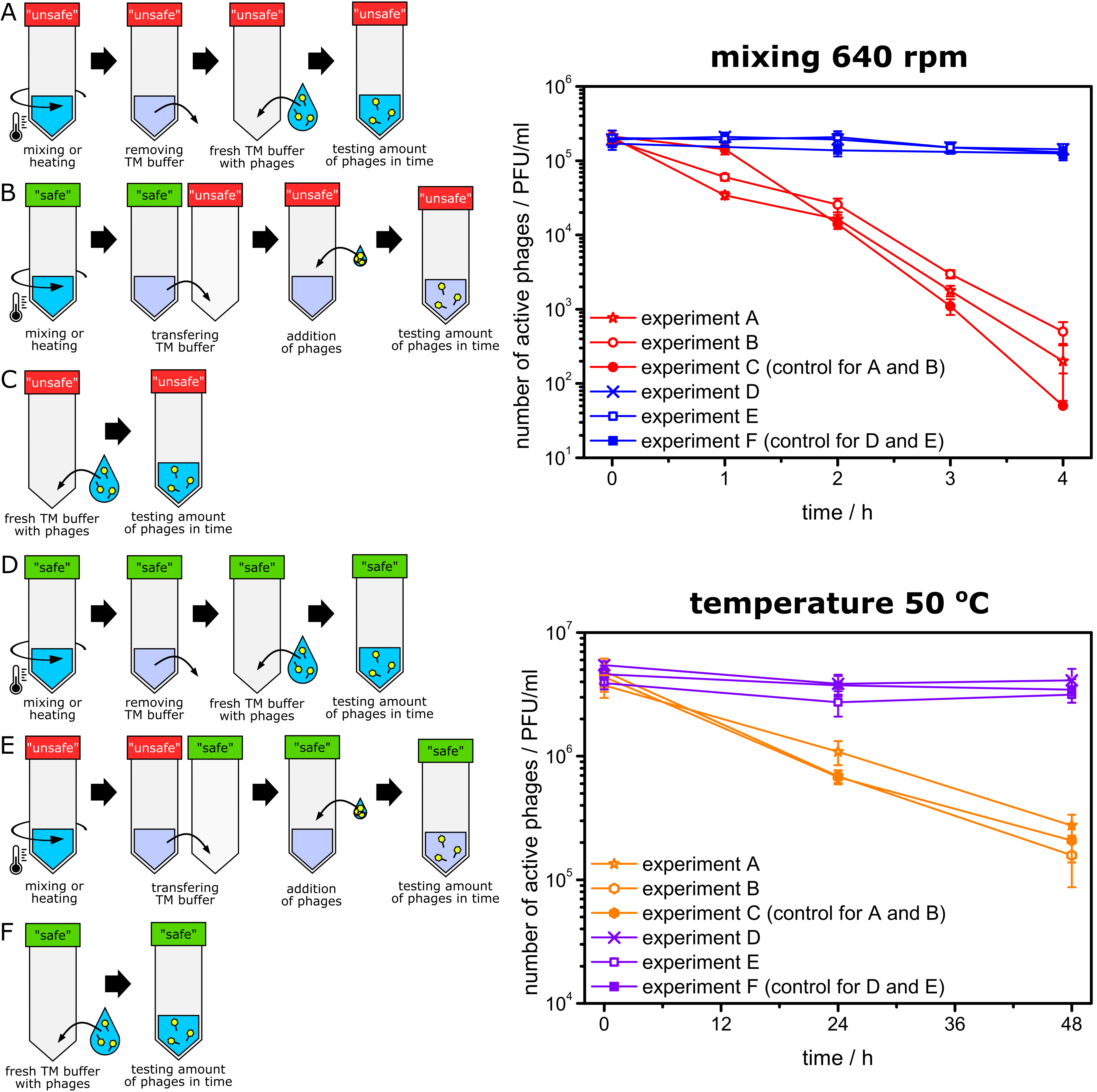


**Figure S6.** Test if any compounds are leaching from plastics and can cause the observed effect of the decrease of the number of active phages. Four types of experiments were performed: **A**) washing out leachables from “unsafe” tube; **B**) transferring leachables from “safe” to “unsafe” tube; **D**) washing out leachables from “safe” tube; **E**) transferring leachables from “unsafe” to “safe” tube. Experiments depicted in **C**) “unsafe” and **F**) “safe” tubes constituted respective controls. We did not succeed in changing the character of the tubes (from “safe” to “unsafe” or vice versa) by any of these procedures.

*Countermeasures*

**B**

**A**


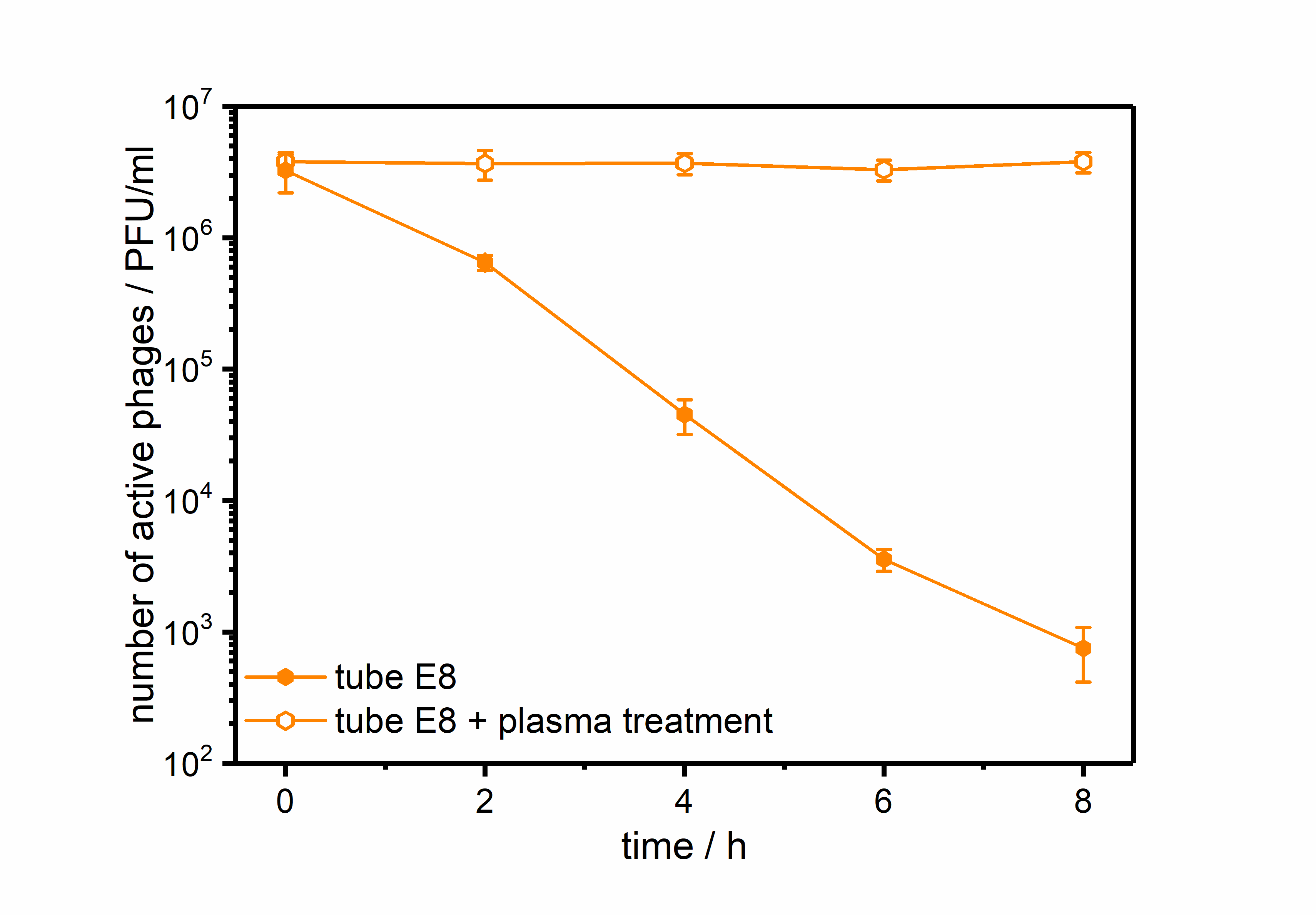

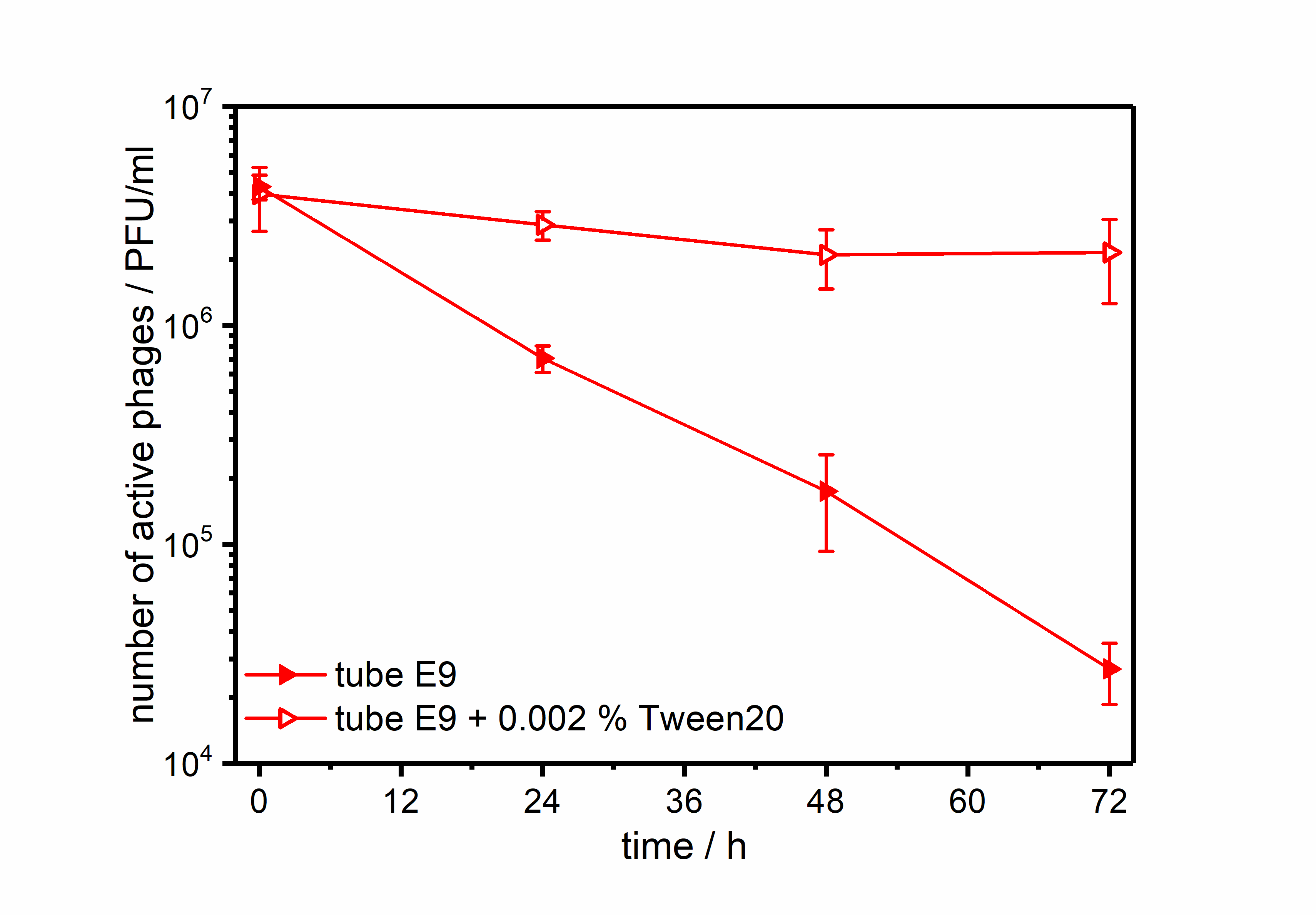


**Figure S7.** Influence of (**A**) plasma treatment (against mixing 800 rpm) of PP tubes and (**B**) addition of 0.002% v/v of Tween20 (against elevated temperature 50 °C) on the adsorption of phages on walls of the “unsafe” (E8, E9) Eppendorf-type tubes.

Both, presence of active phages at the surface of “unsafe” labware in case of lack of phages in the solution (after 5 hours of mixing) and transformation of “unsafe” to “safe” tubes by plasma treatment or addition of Tween20 indicated that the decrease of the number of active phages is due to the interactions of virions with the surface of PP labware. The positive effect of Tween20 and plasma treatment suggested that these interactions depend mainly on the hydrophobicity of the surface.


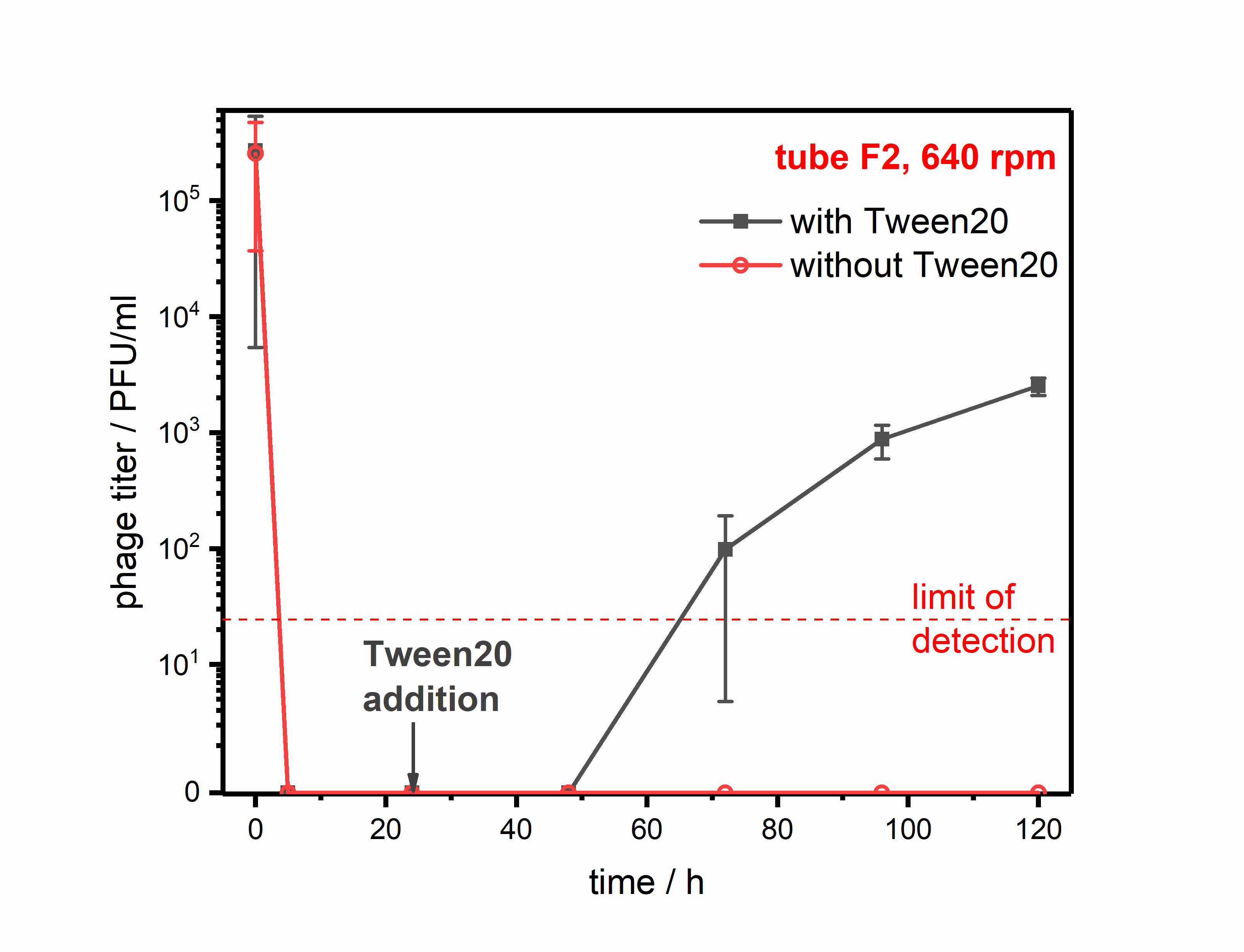


**Figure S8**. Addition of Tween20 results in the reappearance of phages pre-adsorbed on the walls of plastic containers.

*2.4.3. Autoclaving*

Autoclaving is a commonly used method for sterilization of labware. Grześkowiak and Hüble showed that autoclaving has a negative impact on the quality of plastic labware ^43^. We checked whether autoclaving could influence the observed decrease of the number of active phages in suspensions at elevated temperatures. We autoclaved (single or double autoclaving run) “safe” and “unsafe” tubes, and we analyzed the change in the number of active phages over time at 50 °C. We proved that autoclaving of both “safe” (E5) and “unsafe” (E8) tubes does not change the character of the tubes (see **Figure S7** in **Supporting Information)**.


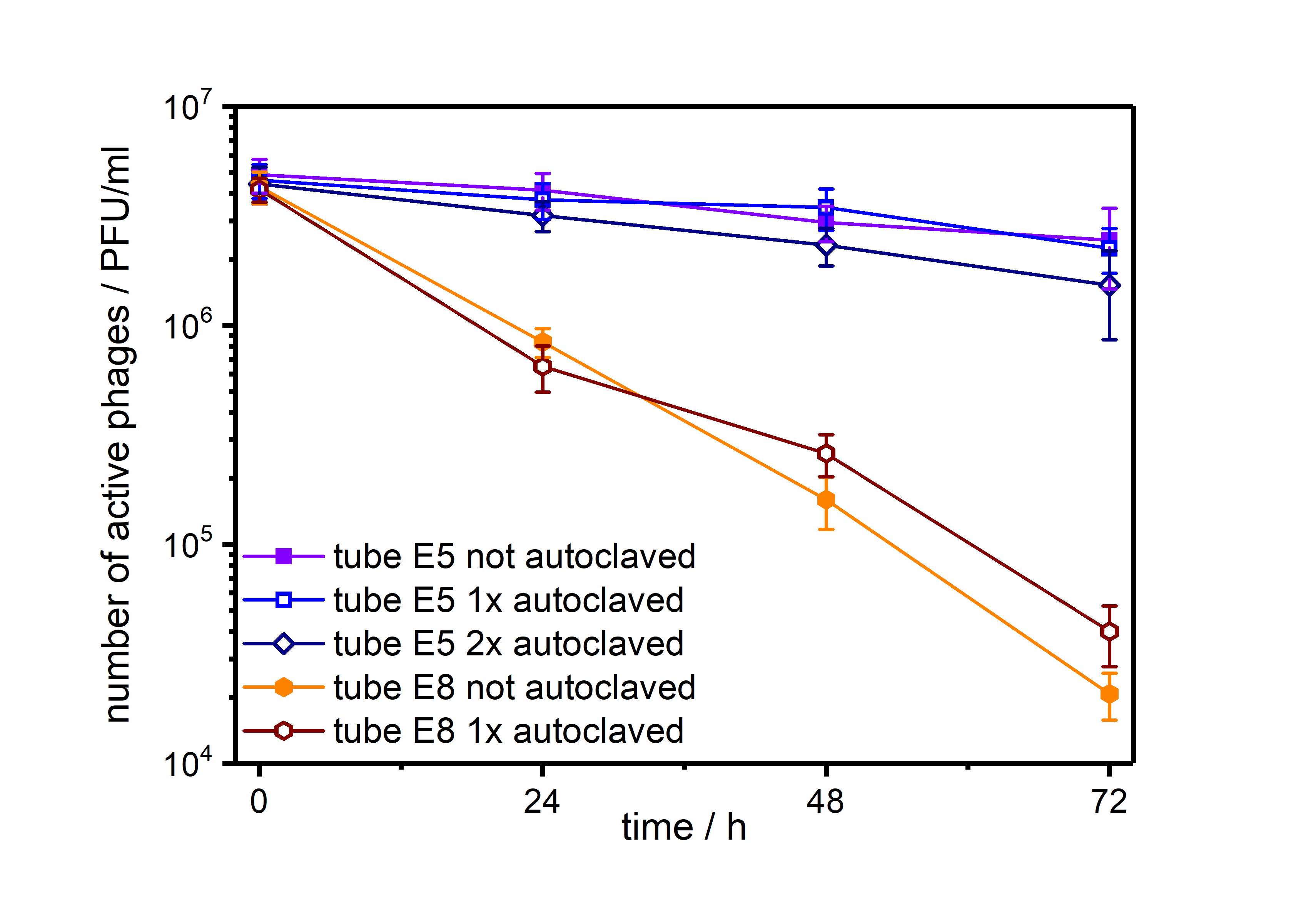


**Figure S9**. Influence of autoclaving of tubes on adsorption of phages on the surface of PP tubes. Phages were kept in 50 °C in both “safe” (E5) and “unsafe” (E8) tubes autoclaved a different number of times.


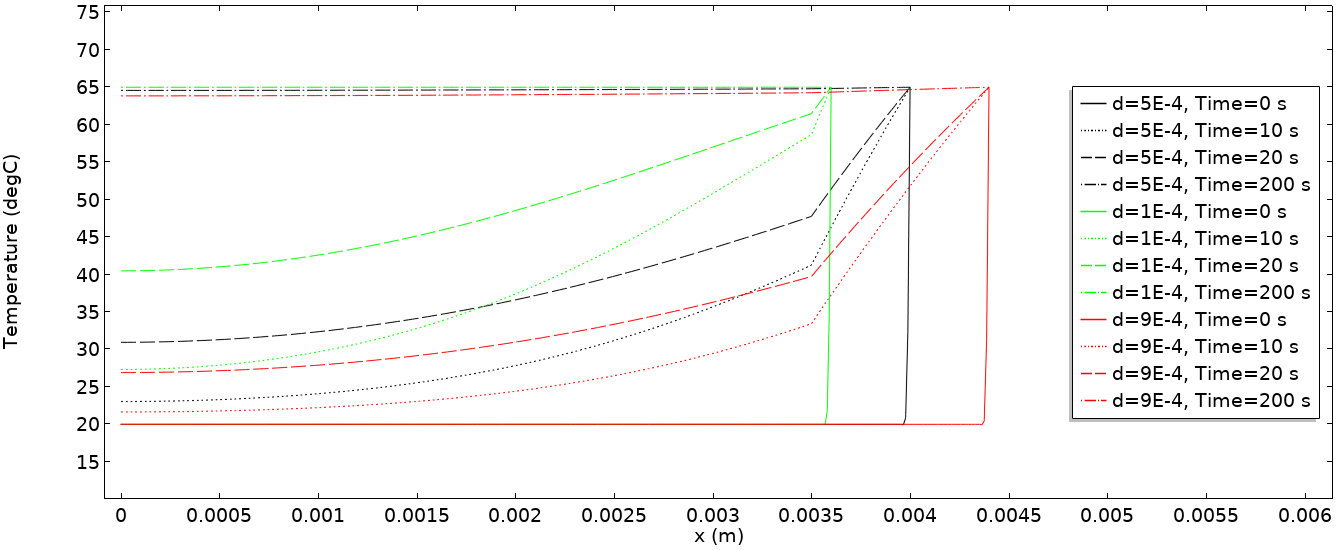


**A**


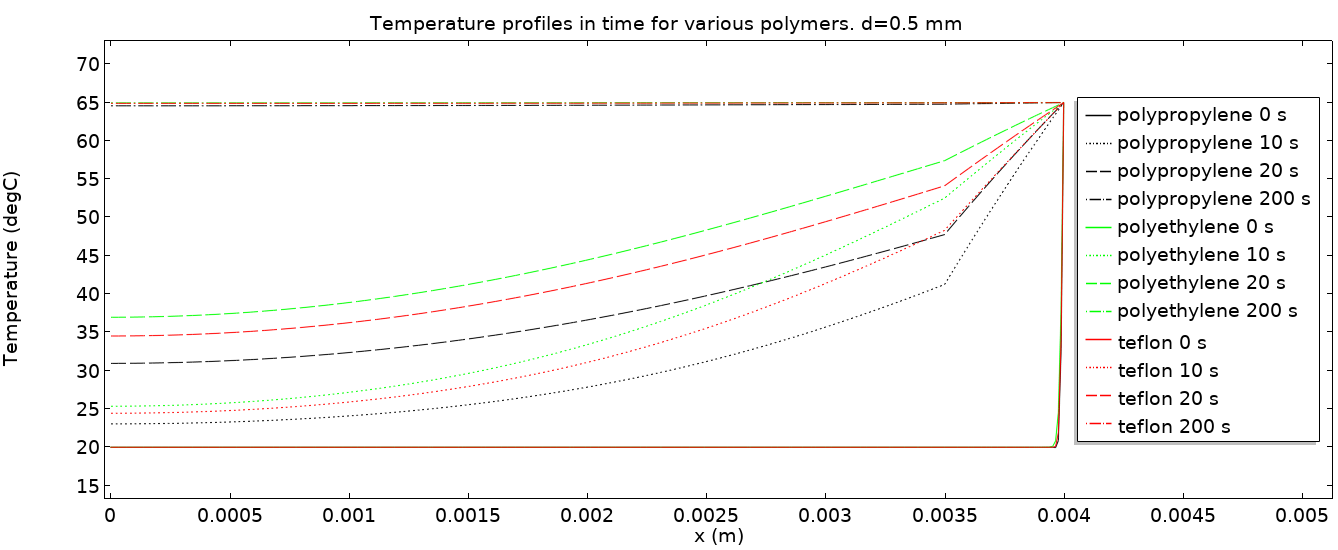


**B**

**Figure S10**. Results of simulation proving that differences in wall thickness (**A**) and intrinsic properties of materials (**B**) are not responsible for differences in observed differences in phage titer in various tubes upon exposure to elevated temperature. In all cases, thermal equilibrium was achieved in all studied cases (thin, regular, and thick walls and polypropylene, polyethylene, and Teflon) in under 200 s, much slower than the observed effect of the decrease of phage titer.

REFERENCES

1. Kuzmanovic, D. A., Elashvili, I., Wick, C., Connell, C. O. & Krueger, S. Bacteriophage MS2 : Molecular Weight and Spatial Distribution of the Protein and RNA Components by Small-Angle Neutron Scattering and Virus Counting. **11**, 1339–1348 (2003).

2. Cuervo, A. & Carrascosa, J. L. Bacteriophages: Structure. *eLS. John Wiley Sons* **224**, 233–240 (2012).

3. McDonald, G. R. *et al.* Bioactive Contaminants Leach from Disposable Laboratory Plasticware. *Science.* **322**, 917–917 (2008).

4. Lee, T. W., Tumanov, S., Villas-Bôas, S. G., Montgomery, J. M. & Birch, N. P. Chemicals eluting from disposable plastic syringes and syringe filters alter neurite growth, axogenesis and the microtubule cytoskeleton in cultured hippocampal neurons. *J. Neurochem.* **133**, 53–65 (2015).

5. Vandenberg, L. N., Hauser, R., Marcus, M., Olea, N. & Welshons, W. V. Human exposure to bisphenol A (BPA). *Reprod. Toxicol.* **24**, 139–177 (2007).
